# The interplay between BAX and BAK tunes apoptotic pore growth to control mitochondrial-DNA-mediated inflammation

**DOI:** 10.1016/j.molcel.2022.01.008

**Published:** 2022-03-03

**Authors:** Katia Cosentino, Vanessa Hertlein, Andreas Jenner, Timo Dellmann, Milos Gojkovic, Aida Peña-Blanco, Shashank Dadsena, Noel Wajngarten, John S.H. Danial, Jervis Vermal Thevathasan, Markus Mund, Jonas Ries, Ana J. Garcia-Saez

**Affiliations:** 1 Interfaculty Institute of Biochemistry, University of Tübingen, 72076 Tübingen, Germany; 2 Department of Biology/Chemistry and Center for Cellular Nanoanalytics (CellNanOs), University of Osnabrück, 49076 Osnabrück, Germany; 3 Institute for Genetics and Cologne Excellence Cluster on Cellular Stress Responses in Aging-Associated Diseases (CECAD), University of Cologne, 50931 Cologne, Germany; 4 Cell Biology and Biophysics Unit, European Molecular Biology Laboratory (EMBL), 69117 Heidelberg, Germany

**Keywords:** BAX, BAK, BCL-2, inflammatory cell death, pore-forming protein, super-resolution microscopy, AFM, single-molecule imaging, mitochondria, membrane pore

## Abstract

BAX and BAK are key apoptosis regulators that mediate the decisive step of mitochondrial outer membrane permeabilization. However, the mechanism by which they assemble the apoptotic pore remains obscure. Here, we report that BAX and BAK present distinct oligomerization properties, with BAK organizing into smaller structures with faster kinetics than BAX. BAK recruits and accelerates BAX assembly into oligomers that continue to grow during apoptosis. As a result, BAX and BAK regulate each other as they co-assemble into the same apoptotic pores, which we visualize. The relative availability of BAX and BAK molecules thereby determines the growth rate of the apoptotic pore and the relative kinetics by which mitochondrial contents, most notably mtDNA, are released. This feature of BAX and BAK results in distinct activation kinetics of the cGAS/STING pathway with implications for mtDNA-mediated paracrine inflammatory signaling.

## Highlights

• BAK oligomerizes faster into smaller lines, arcs, and rings than those formed by BAX
• BAK recruits BAX to co-assemble into the same supra-molecular apoptotic structures
• BAX and BAK reciprocal regulation defines the growth dynamics of apoptotic pores
• BAX and BAK have distinct roles in modulating the inflammatory outcome of apoptosis

## Graphical abstract

**Figure:**
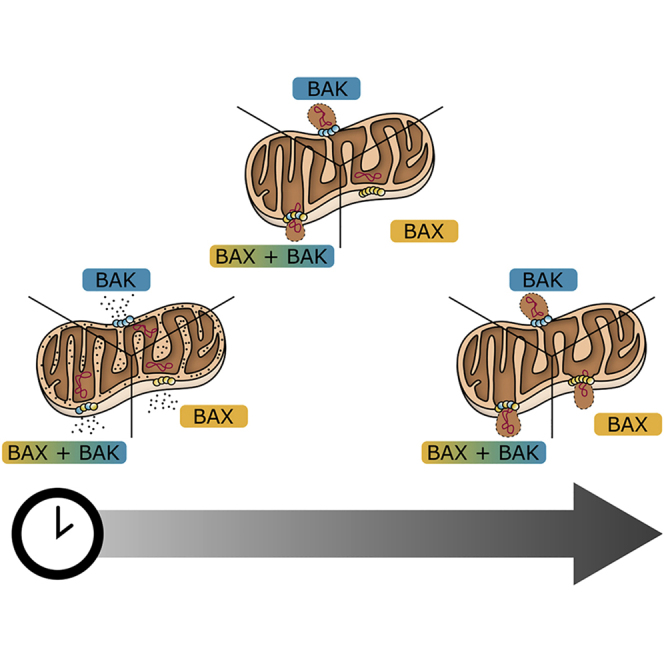

## Teaser

BAX and BAK mediate membrane permeabilization in the mitochondrial pathway of apoptosis. Cosentino et al. show that the growth rate and permissiveness to mtDNA of the apoptotic pore can be dynamically modulated by the balance between BAX and BAK molecules, which regulate each other’s assembly, thereby impacting downstream inflammatory signaling.

## Introduction

BAX and BAK are proapoptotic members of the BCL-2 family required for the permeabilization of the mitochondrial outer membrane (MOM) that during apoptosis releases apoptogenic factors such as cytochrome *c* and Smac/DIABLO into the cytosol to unleash the apoptotic cascade (Czabotar et al., 2014; Cosentino and García-Sáez, 2017). Cells from BAX and BAK double-knockout (DKO) mice are resistant to most apoptotic stimuli, and the integrity of their MOM is maintained even upon overexpression of proapoptotic BH3-only proteins (Wei et al, 2001). Furthermore, the phenotype of BAX/BAK DKO mice is perinatal lethal, and the few mice that reach adulthood present defective tissue homeostasis due to reduced apoptosis (Lindsten et al., 2000). Despite the interest in BAX and BAK as pharmaceutical targets for cancer therapy, our insufficient understanding of their molecular mechanism has so far limited the successful development of small molecule drugs.

BAX and BAK exist in an inactive form in healthy cells and shuttle between cytosol and mitochondria in a retro-translocation cycle promoted by anti-apoptotic BCL-2 proteins (Edlich et al., 2011; Schellenberg et al., 2013; Todt et al., 2015). During apoptosis, they accumulate at discrete sites at mitochondria, undergo a conformational change, and oligomerize concomitant with MOM permeabilization (MOMP) (Cosentino and García-Sáez, 2017). We previously reported that BAX oligomers exist in model membranes as multiple species, mostly based on dimer units (Subburaj et al., 2015). Super-resolution imaging revealed that BAX organizes into lines, arcs, and rings, with both arcs and rings being able to directly perforate lipid membranes (Große et al., 2016; Salvador-Gallego et al., 2016). Recent studies also showed that, after MOMP, BAX and BAK form large megapores that release big macromolecules such as mitochondrial DNA (mtDNA) (McArthur et al., 2018; Riley et al., 2018). Once in the cytosol, mtDNA triggers an inflammatory response through the cyclic GMP-AMP synthase/stimulator of interferon genes (cGAS/STING) signaling pathway that is normally dampened by caspase activity (Rongvaux et al., 2014; White et al., 2014; Giampazolias et al., 2017). These findings connect BAX and BAK to the regulation of the inflammatory outcome of apoptosis.

BAX and BAK exhibit high homology in sequence and structurally (Westphal et al., 2014; Czabotar et al., 2014), which, together with the need to knock out both proteins to block MOMP, has led to the assumption that both proteins have fully overlapping functions and share their molecular mechanism. Despite their functional redundancy, BAX and BAK differ in certain aspects. For example, their inactive forms present different steady-state cellular distribution, with BAX mainly cytosolic and BAK mainly mitochondrial. They are also usually expressed at different levels and most cell types contain higher amounts of BAX than of BAK (https://www.proteomicsdb.org/proteomicsdb; BAX ID Q07812, BAK ID Q16611). In addition, they seem to have distinct preferential binding to different BH3-only proteins and to be differentially activated by them (Kale et al., 2018; Singh et al., 2019; Sarosiek et al., 2013). Today, we still fail to understand whether these differences support a distinct molecular mechanism of action of BAX versus BAK and which biological implications this may have.

Here, we combined different single-molecule microscopy approaches to analyze the supra-molecular organization of BAX and BAK at the nanoscale. Using single-molecule localization microscopy (SMLM), we found that in the mitochondria of apoptotic cells BAK assembles into lines, arcs, and rings that are significantly smaller and more narrowly distributed compared with those of BAX. This is supported by single particle stoichiometry analysis of BAK and BAX oligomers and by atomic force microscopy (AFM) in model membranes revealing that BAK rings and arcs associate with membrane pores. Comparative analysis of the growth rate of single BAX and BAK clusters in cells undergoing apoptosis showed that distinct assembly properties underlie these differences and that BAX and BAK modulate each other’s oligomerization. Mechanistically, the interplay between BAX and BAK is based on BAK promoting BAX recruitment and activation followed by their co-assembly into the same supra-molecular structures, as directly visualized by stimulated emission depletion (STED) microscopy. This has functional implications since the balance between BAX and BAK molecules regulates the dynamics of apoptotic pore growth, and thereby the relative kinetics of Smac and mtDNA release from mitochondria, which affects the downstream activation of the cGAS/STING signaling pathway and of bystander T cells. Our results support a model in which the regulation of the apoptotic pore growth by the interplay between BAX and BAK tunes the immunogenic impact of apoptosis.

## Results

### BAK assembles into line-, arc-, and ring-like structures in apoptotic cells

**Figure 1:**
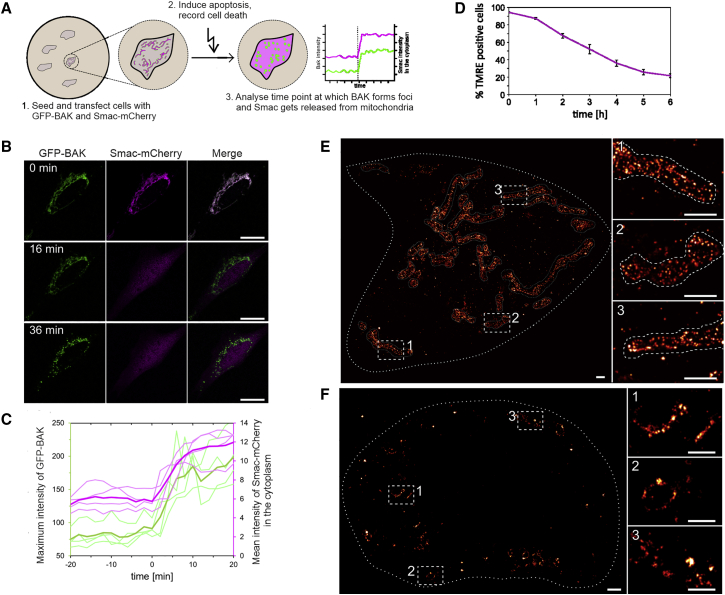
BAK assembly into distinct structures correlates with mitochondrial outer membrane permeabilization (A) Scheme of the assay to monitor Smac release (magenta) from mitochondria to the cytosol due to mEGFP-BAK (green) foci formation. (B) Representative confocal images of a BAX/BAK DKO HCT116 cell transfected with mEGFP-BAK (green) and Smac-(1-60)-mCherry (magenta) before (0 min) and 16 and 36 min after apoptosis induction with 1-μM STS. Scale bars, 10 μM. (C) Maximum fluorescent intensity of mEGFP-BAK (green) and mean fluorescent intensity of cytosolic Smac-(1-60)-mCherry (magenta) for individual cells as in (B) (n = 4). Time point 0 min corresponds to the normalized time point of Smac-mCherry release. The release of Smac-mCherry in the cytosol correlates with an increase in the mEGFP-BAK intensity, which is indicative of foci formation. Bold lines represent the mean of individual dataset (light lines). (D) Percentage of TMRE positive BAX/BAK DKO HCT116 cells transfected with mEGFP-BAK after treatment with 1 μM STS (n = 2). Error bars represent the SD. (E and F) Representative SMLM images of mEGFP-BAK in BAX/BAK DKO HCT116 cells in healthy (E, mitochondria profile is defined by the dotted lines) and apoptotic conditions (F). Right panels are zoomed regions (indicated by white boxes in the main image). Scale bars: 1 μM in the main images and 500 nm in zoomed regions.

To visualize the structural organization of BAK at the nanoscale, we used SMLM as previously with BAX (Salvador-Gallego et al., 2016). We first characterized the dynamics of apoptosis progression under our experimental conditions in BAX/BAK DKO HCT116 cells by live-cell confocal imaging (Figure 1A). Inactive monomeric enhanced GFP-BAK (mEGFP-BAK) appeared homogeneously distributed along the mitochondrial network of untreated cells. Upon apoptosis induction, mEGFP-BAK redistributed into discrete foci at mitochondria, in correlation with Smac-mCherry release into the cytosol as a proxy for MOMP (Figures 1A–1C). These results also confirmed that BAK tagged with mEGFP retained its apoptotic activity. Since apoptosis is a dynamic and generally irreversible process, we fixed cells for SMLM at the time point when 50% cells had undergone MOMP (3 h; Figure 1D).

**Figure 2:**
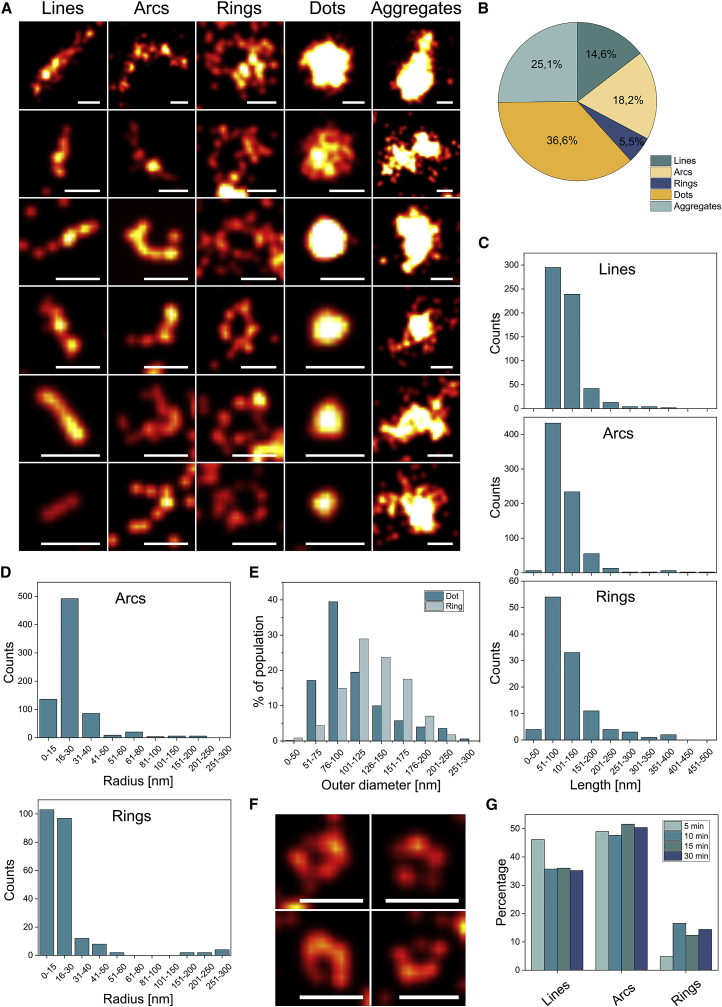
Super-resolution microscopy reveals nanoscopic BAK structures in apoptotic cells (A) Gallery of BAK structures in apoptotic BAX/BAK DKO HCT116 cells transfected with mEGFP-BAK and labeled with an anti-GFP Alexa Fluor 647 nanobody. Scale bars, 100 nm. (B) Relative distribution of the imaged BAK structure types over the total number of structures in all measured cells (n = 12). (C and D) Quantification of the line and arc length and of the ring perimeter (C) and of the arc and ring radius (D) for all analyzed cells (n = 12). (E) Quantification of the diameter of dots (blue) and outer diameter of rings (light blue) for all analyzed cells (n = 12). (F) Exemplary images representing dots with less intensity in the center. Scale bars, 100 nm. (G) Relative distribution of all collected line (minimum 84), arc (minimum 112), and ring (minimum 24) structures (cells n = 12) at different time points after first foci appearance. See also Figures S1 and S2.

SMLM of BAK in healthy cells confirmed its homogeneous distribution on the mitochondrial network, typically appearing as individual molecules lining the MOM in super-resolution images (Figure 1E). In contrast, in apoptotic cells BAK reorganized into discrete foci on fragmented mitochondria with distinct macromolecular architectures (Figure 1F), which we quantified with ASAP (automated structures analysis program [Danial and Garcia-Saez, 2019]). While most of the signal appeared as dots and aggregates, roughly 40% of BAK assemblies corresponded to lines, arcs, and rings of similar length and radius, independently of the cell line (Figures 2 and S1). These findings revealed that the architecture of BAK clusters in apoptotic cells was similar to that of BAX (Salvador-Gallego et al., 2016; Große et al., 2016), but, to our surprise, BAK formed smaller assemblies (with a mean ring radius of 18 nm compared with 34 nm of BAX [Salvador-Gallego et al., 2016]), which also were more homogeneous in size under similar conditions (Figures 2C, 2D, and S1D).

Given that 2D SMLM does not take into account the 3D nature of mitochondria and the relative orientation of the BAK structures, we cannot exclude that a fraction of the dots and aggregates correspond to unresolved distinct architectures. For example, some dots with size comparable to that of rings presented lower fluorescence in the center (Figures 2E and 2F), suggesting that they might be unresolved rings.

The finding that lines, arcs, and rings had a similar average length raised the question whether lines and arcs were intermediate structures evolving to a final ring. To study the temporal evolution of BAK structures, we established correlative live-cell confocal and SMLM microscopy (Figure S2). Using this approach, we analyzed single cells at different time points after MOMP and compared the distribution of BAK assemblies. We detected a slight decrease of linear structures accompanied by an increase in rings shortly after BAK foci formation, which then remained stable (Figure 2G). This indicated that the evolution of BAK structures occurs within the first 10 min of foci formation. The modest accumulation of rings at the expense of lines suggests that a fraction of BAK structures may develop from lines to rings. Although we cannot discard that some of the lines and arcs correspond to ring structures with tilted orientation with respect to the observation plane, the persistence of a high percentage of lines and arcs suggests that they are stable structures likely representing distinct entities.

### Both arcs and rings of BAK are able to form membrane pores of a smaller size than BAX

**Figure 3:**
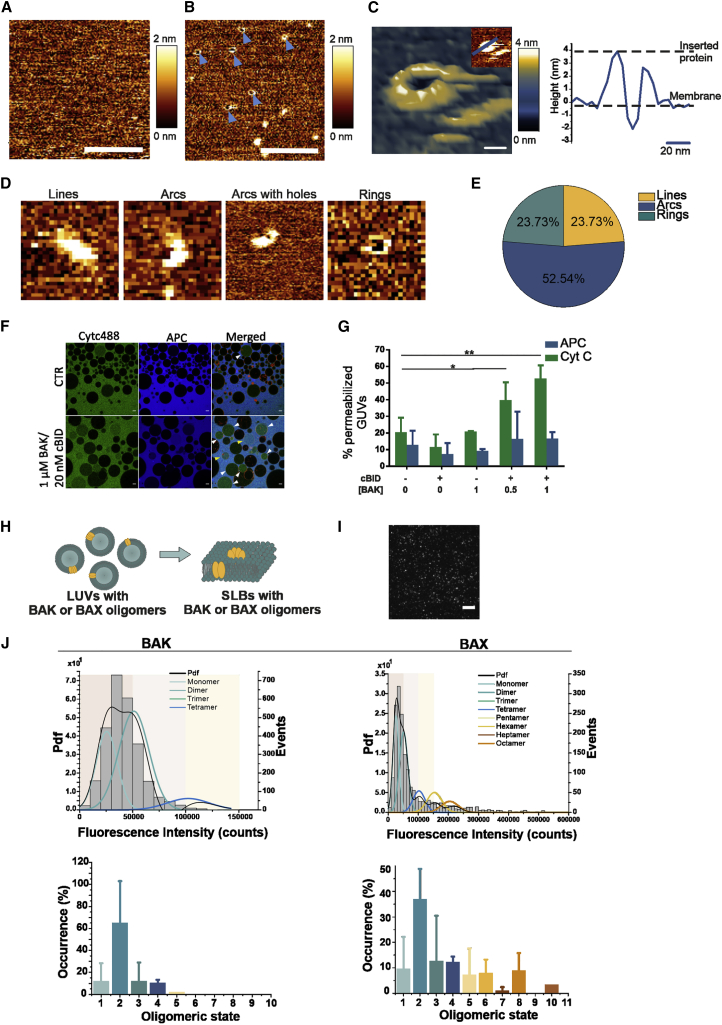
BAK assemblies are associated with stable membrane pores smaller than BAX (A and B) Representative image of an EPC:CL (80:20 mol %) membrane without (A) and with (B) BAK pores (indicated by the blue arrows) imaged by atomic force microscopy (AFM). Scale bars, 200 nm. (C) 3D representative image of a BAK pore (left) and its height profile (right, corresponding to the blue line in the 2D image inset). Scale bars, 20 nm. (D) Representative BAK structures imaged by AFM. Picture size 100 nm. The full-color height range of the topographs from low (brown-orange) to high (yellow-white) is 2 nm. (E) Percentage distribution of each BAK structure types over the total number of analyzed structures (lines = 14, arcs = 31, and rings = 14). All data (A–E) were obtained from at least four independent experiments. (F) Representative images of giant unilamellar vesicles (GUVs, black and red membrane in the merged channel) of EPC:CL (80:20 mol %) lipid composition without (top) or with (bottom) incubation with cBid-activated BAK. After 1-h incubation, Cytc488 (green) and APC (blue) were added and images were taken 30 min later. White and yellow arrow heads indicate Cytc488 and Cytc488-APC permeabilized GUVs, respectively. Scale bars, 10 μm. (G) Percentage of GUVs internalizing the fluorescent probes at different concentrations of BAK and 20 nM of cBid. Data (F and G) were obtained from at least three independent experiments and a minimum of 50 vesicles were analyzed per condition. Error bars represent the SD. ^∗^p < 0.05; ^∗∗^p < 0.01 (one-way ANOVA with Dunnett's multiple comparison test). (H) Schematic representation of the protocol used for sample preparation of stoichiometry experiments in model membranes (BAX and BAK depicted in yellow, see STAR Methods). (I) Representative TIRF image of an SLB containing BAK oligomers (bright spots). Scale bars, 10 μm. (J) Upper panel: representative fluorescence intensity distribution of BAK-488 and BAX-488 oligomers (minimum 1,500 particles per condition) obtained from SLB samples with 10 nM of protein. The obtained brightness distribution was plotted as a probability density function (Pdf, black) or, alternatively, as a histogram, and fitted with a linear combination of Gaussians to estimate the percentage of occurrence of particles containing n-mer-labeled molecules (see color code in the graph). The three panels in the background indicate each a 50,000 counts range and are for a visual comparison between BAX and BAK intensity distribution graphs. Lower panel: percentage of occurrence of BAK-488 and BAX-488 different oligomeric species calculated as the average value from two different experiments. Data provided are corrected for partial labeling (see STAR Methods). The error bars correspond to the SD from the different experiments. See also Figures S3 and S4.

To functionally associate the nano-assemblies of BAK detected in apoptotic cells by SMLM with their ability to form membrane pores, we used AFM. We produced recombinant full-length monomeric BAK (Figures S3A–S3D; Leshchiner et al., 2013) and prepared supported lipid bilayers (SLBs) from proteoliposomes containing activated BAK. While control membranes appeared flat and smooth in AFM images (Figure 3A), SLBs containing activated BAK presented structures protruding from the membrane (Figures 3B and 3C). Remarkably, besides undefined aggregates, these structures also included lines, arcs, and rings (Figures 3D and S3E). The identification of lines and arcs by AFM, in which the membrane plane has a constant orientation, supports the existence of these structures also in apoptotic cells, as suggested by the SMLM data.

Importantly, the 3D topography of the membrane surface provided by AFM allowed the visualization of membrane pores associated with both rings and arcs of BAK (Figures 3C, 3D, and S3E). Compared with BAX pores observed by AFM (Salvador-Gallego et al., 2016), BAK pores were smaller and more homogeneous, with an average pore radius of 8.12 ± 3.03 nm for the rings (Figure S3F). Due to their small size, we estimate that a fraction of them could not be resolved by our setup and appeared as aggregates on the membrane.

To estimate the size of BAK pores with an alternative method, we measured the influx of external, fluorescently labeled cytochrome *c* (12.5 kDa) and allophycocyanin (APC, 104 kDa) into giant unilamellar vesicles (GUVs) at different BAK concentrations. While cytochrome *c* was promptly internalized into GUVs in a concentration-dependent manner, we did not detect internalization of APC under our experimental conditions (Figures 3F and 3G). These results indicate that full-length BAK formed stable pores in GUVs smaller (or in the same range) than APC, in contrast to previous observations with BAX and with a truncated version of BAK (Bleicken et al., 2013a). Our findings in model membranes show the same trend as the SMLM data, collectively demonstrating that BAK forms smaller pore structures than BAX.

### Oligomerization of BAX, but not BAK, is sensitive to protein density in the membrane

The distinct size of the structures formed by BAX and BAK assemblies in the membrane raised the question of whether they exhibit different oligomerization properties. To address this matter, we compared the stoichiometry *in vitro* of membrane-bound BAX and BAK oligomers at different concentrations by TIRF single-molecule imaging as in Subburaj et al. (2015) (Figures 3H–3J and S4). We produced functional mutants of BAX and BAK fluorescently labeled at single cysteines (see STAR Methods). Unexpectedly, the brightness of the individual BAX-488 particles, but not BAK-488 particles, in the population appeared as a broad distribution (Figures 3J and S4A), typical of multiple co-existing oligomeric species in the membrane. We then estimated the number of BAK and BAX molecules in the individual clusters based on the average intensity of their respective monomers. As with BAX, monomeric BAK-488 was obtained by adding it directly on an SLB (Subburaj et al., 2015) due to unspecific interactions with the glass support preventing BAK oligomerization (Figures S4C–S4E). In contrast, BAK-488 molecules pre-incubated with liposomes assembled into oligomers, predominantly dimers (Figures 3J, S4A, and S4B), independently of the activation strategy (Figures S4F and S4G). In addition, at the nanomolar range of concentrations required for TIRF single-molecule imaging, the distribution of BAX-488, but not BAK-488, oligomers increased with protein concentration. Compared with BAK, BAX-488 showed higher fluorescence intensity distributions at all tested concentrations and reached higher oligomeric states up to 10-mer (Figures 3J, S4A, and S4B). These data suggest that BAK has a lower tendency than BAX to oligomerize in the membrane, in line with the formation of smaller structures observed by AFM (Figures 3A–3E). Furthermore, BAX but not BAK oligomerization strongly depends on membrane density.

### Real-time analysis of BAX and BAK assembly in the mitochondria of apoptotic cells reveals different oligomerization properties

**Figure 4:**
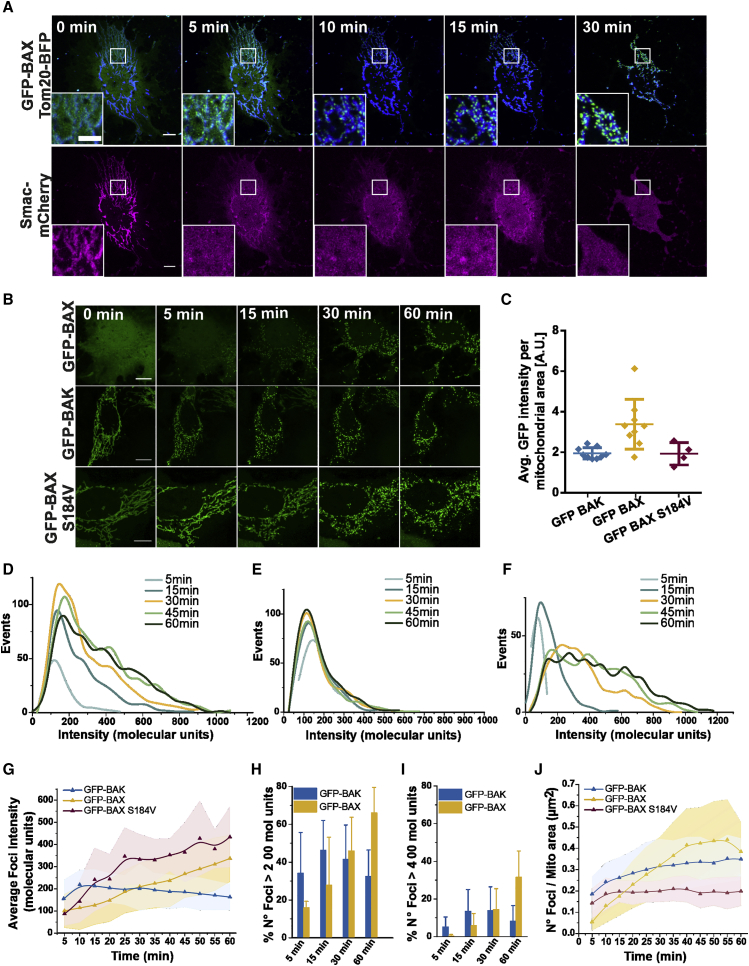
BAX and BAK foci assembly proceeds via a different mechanism of oligomerization (A) Representative confocal images of a BAX/BAK DKO U2OS cell transfected with mEGFP-BAX (green), Tomm20-BFP (blue) and Smac-1-60-mCherry (magenta) and treated with ABT-737, S63845 and qVD-Oph for apoptosis induction. Time points are after Smac-1-60-mCherry release from mitochondria. Scale bars, 10 μm. Inset: apoptotic foci of mEGFP-BAX growing over time on mitochondria. Scale bars, 3 μm. (B) Representative photon-counting confocal images of BAX/BAK DKO U2OS cells transfected with mEGFP-BAK, mEGFP-BAX, or mEGFP-BAX(S184V) and treated for apoptosis induction. Scale bars, 10 μm. (C) Average mEGFP expression level per mitochondrial area in individual cells (represented by individual dots in the plot) transfected with mEGFP-BAK, mEGFP-BAX, or mEGFP-BAX(S184V) detected by single-cell fluorescent intensity analysis of mEGFP signal. The error bars correspond to the SD from the different experiments. (D–F) Representative distribution of foci intensity, in stoichiometric units, at different time points in individual apoptotic cells overexpressing mEGFP-BAX (D), mEGFP-BAK (E), or mEGFP-BAX(S184V) (F). (G) Average foci intensity obtained from the fitting of foci distributions as in (D–F) at each time point for individual apoptotic cells expressing mEGFP-BAX (yellow, n = 9), mEGFP-BAK (blue, n = 9) or mEGFP-BAX(S184V) (purple, n = 4). Lines in the graph correspond to the average values from all measured cells and colored areas correspond to data variability from single cells (mean ± SD). (H and I) Number of foci with molecularity higher than 200 (H) or 400 (I) normalized to the total number of foci in the cell at different time points after Smac release. Average total n° foci over analyzed cells is ranging from 67 (at 5 min) to 517 (60 min) for mEGFP-BAX (n = 9 cells) and from 250 (at 5 min) to 481 (60 min) for mEGFP-BAK (n = 9 cells). The error bars correspond to the SD from the different experiments. (J) Number of foci per mitochondrial area over time for apoptotic cells as in (G). See also Figures S5 and S6.

We hypothesized that the structural differences between BAX and BAK oligomers in the membrane could be the result of mechanistic differences in their assembly. To address this question in the more physiological setting of the cell, we implemented a method to quantify the real-time kinetics of BAX and BAK oligomerization in mitochondria of apoptotic cells that allows estimating their stoichiometry over time (see STAR Methods and Figure 4). Based on photon-counting confocal microscopy in combination with ratiometric analysis (Figure S5; Jenner et al., 2020; Finan et al., 2015; Verdaasdonk et al., 2014), this approach compares the fluorescence intensity of the protein complex of interest with a standard of known stoichiometry labeled with the same fluorophore. As a standard, we used the 32-mer mEGFP-tagged nuclear pore complex component 96 (NUP96), endogenously expressed in the same U2OS cell type used for our experiments (Figure S5; Thevathasan et al., 2019).

Figure 4A shows that the assembly of transfected mEGFP-BAX into foci at mitochondria of BAX/BAK DKO U2OS cells correlated with Smac-mCherry release into the cytosol, indicative of MOMP (see also Figures S6A and S6B). We then monitored the growth of individual foci of mEGFP-BAX or mEGFP-BAK over time until the point when the mitochondrial network was too fragmented and the foci started to coalesce due to cell shrinkage (Figure 4B). Since MOMP takes place in the individual cells of the population at different time points after treatment, we synchronized the analysis of the kinetics of mEGFP-BAX and mEGFP-BAK assembly by setting Smac-mCherry release as a temporal reference. We determined the stoichiometry of the individual foci at each time point for BAX/BAK DKO cells expressing mEGFP-BAX or mEGPF-BAK at similar levels (Figure 4C). We found that the fluorescence intensities of mEFGP-BAX oligomers increased over time into a broad distribution, ranging from tens to several hundreds of units (Figure 4D). In contrast, the fluorescence intensities of mEGFP-BAK foci did not significantly change after 10 min from MOMP and displayed a more uniform oligomer distribution centered around 100–200 U (Figure 4E). This difference in the size of the BAX and BAK clusters is in good agreement with the results from SMLM and reconstituted systems.

By plotting the average foci stoichiometry over time, we observed that mEGFP-BAK oligomerized faster and reached a stable size of around 200 molecules a few minutes after MOMP (Figure 4G). In contrast, the assembly of mEGFP-BAX foci was slower, and it took around 30 min to reach the same average number of units as mEGFP-BAK under these experimental conditions (Figure 4G). Remarkably, the average stoichiometry of mEGFP-BAX continued to increase during the measurement time, resulting in a steady increase in the number of large foci (containing more than 200 or 400 U) (Figures 4H and 4I). In contrast to mEGFP-BAK, the mitochondrial density of mEGFP-BAX foci also increased with time (Figure 4J). These results were independent of the cell line and treatment (Figures S6C–S6H).

To confirm that these differences were not due to the different initial cellular localization of BAX and BAK or to slightly different expression levels, we analyzed a mutant of BAX, BAX(S184V), which retains activity but localizes constitutively at mitochondria (Verdaasdonk et al., 2014) and is expressed at similar levels as BAK (Figures 4B and 4C). mEFGP-BAX(S184V) assembled faster than mEFGP-BAX, but slower than mEGFP-BAK, and still arranged into a broad distribution of oligomers whose molecularity steadily increased similar to BAX (Figure 4F). These results indicate that the faster assembly kinetics of BAK is only partially due to its mitochondrial localization and that the saturation in BAK oligomers’ size is not due to molecular availability. Collectively, these findings demonstrate that the assembly of BAX and BAK upon their activation in apoptosis is mechanistically different.

### BAK and BAX modulate each other’s oligomerization by co-assembling into the same supra-molecular structures

**Figure 5:**
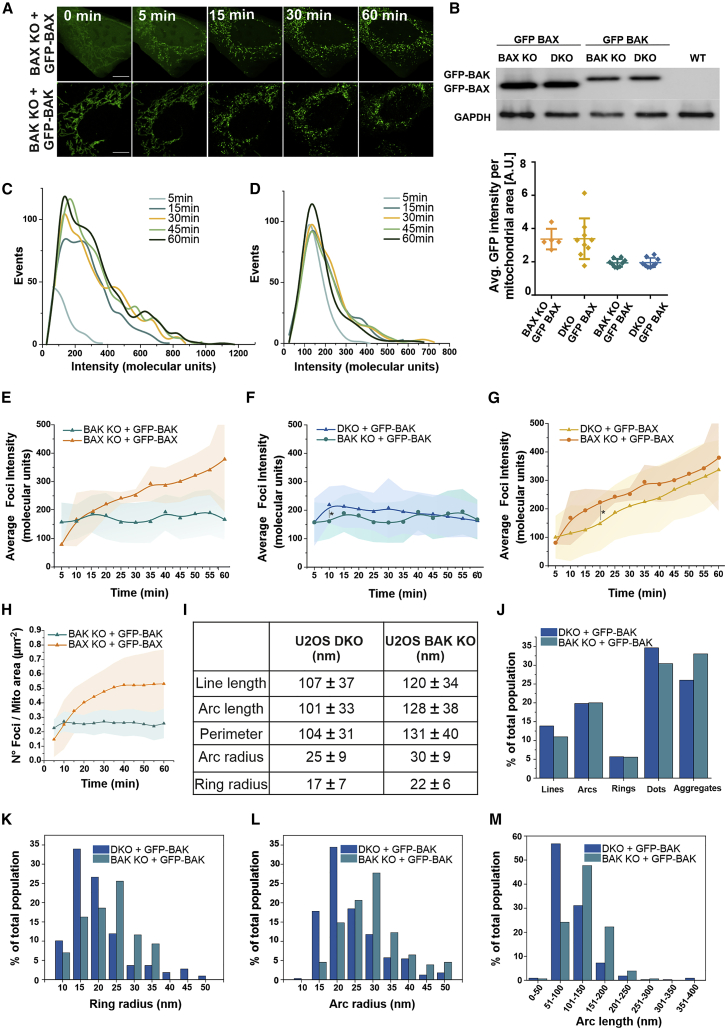
Reciprocal contribution of BAK and BAX to their assembly kinetics and molecularity (A) Representative photon-counting confocal images of BAX/BAK KO U2OS cells transfected with mEGFP-BAX or mEGFP-BAK, respectively, after apoptosis induction by ABT-737, S63845, and qVD-OPh. Time points are after Smac-mCherry release. Scale bars, 10 μm. (B) Comparison of the expression levels of mEGFP-BAK and mEGFP-BAX in single or double BAK/BAX KO cells detected by western blot (upper) or by single-cell (individual dots in the plot) fluorescent intensity analysis of mEGFP signal (bottom). WT, wild type.The error bars correspond to the SD from the different experiments. (C and D) Representative foci intensity distribution, in stoichiometric units, at different time points in an apoptotic BAX (C) and BAK (D) KO cell expressing mEGFP-BAX or mEGFP-BAK, respectively. (E) Average foci intensity obtained from the fitting of foci distributions as in (C and D) at each time point for individual apoptotic cells expressing mEGFP-BAX (orange, n = 5) or mEGFP-BAK (green, n = 9). (F and G) Comparison between the average foci intensity of mEGFP-BAK in BAX/BAK DKO (blue, n = 9) and BAK KO (green, n = 9) cells over time (F) and of mEGFP-BAX in BAX/BAK DKO (yellow, n = 9) and BAX KO (orange, n = 5) cells over time (G). ^∗^p < 0.1 (unpaired t test with Welch’s correction). (H) Number of mEGFP-BAX (orange, n = 5) and mEGFP-BAK (green, n = 9) foci per mitochondrial area in single KO cells over time (total number of events per cell as in C and D). For (E–H), lines in the graph correspond to the average values from all measured cells and colored areas correspond to data variability from single cells (mean ± SD). (I) Average size of the different structural parameters for mEGFP-BAK assemblies in BAX/BAK DKO (n = 7) and BAK KO (n = 6) cells measured by SMLM. Error corresponds to SD. (J) Distribution of the different BAK structure types found in BAX/BAK DKO (n = 7) and BAK KO (n = 6) cells (minimum number of analyzed structure type = 86). (K–M) Comparison of the quantifications of the ring radius (K), arc radius (L), and arc length (M). Number of analyzed structures in single and double KO cells, respectively: 86 and 109 (K), 310 and 363 (L), and 310 and 363 (M).

Since BAX and BAK co-localize into apoptotic foci (Zhou and Chang, 2008), yet they present distinct oligomerization properties, we hypothesized that BAX and BAK may influence each otheŕs assembly. To tackle this question, we quantified the real-time foci stoichiometry of mEGFP-BAX or mEGFP-BAK in single knockout (KO) U2OS cells containing either only endogenous BAK (BAX KO) or only endogenous BAX (BAK KO) (Figures 5A–5D). Compared with the BAX/BAK DKO cells, both mEGFP-BAX and mEGFP-BAK maintained their assembly behavior in the single KO background (Figures 4G, 5E, and 5H), with the remarkable difference that mEGFP-BAX oligomerized faster and mEGFP-BAK slower (Figures 5F and 5G). A comparative analysis of super-resolved BAK structures in the presence or absence of BAX at 30 min after MOMP (Figures 5I–5M) also showed that the presence of endogenous BAX caused a significant increase in their size. The average size of transfected mEGFP-BAK nano-structures in single BAK KO cells was intermediate between that of BAK and BAX in DKO cells, while their shape and distribution was not affected. Altogether, these findings reveal that BAX and BAK reciprocally contribute to each other's assembly: BAX increases the size of BAK apoptotic structures, while BAK accelerates BAX assembly kinetics.

**Figure 6:**
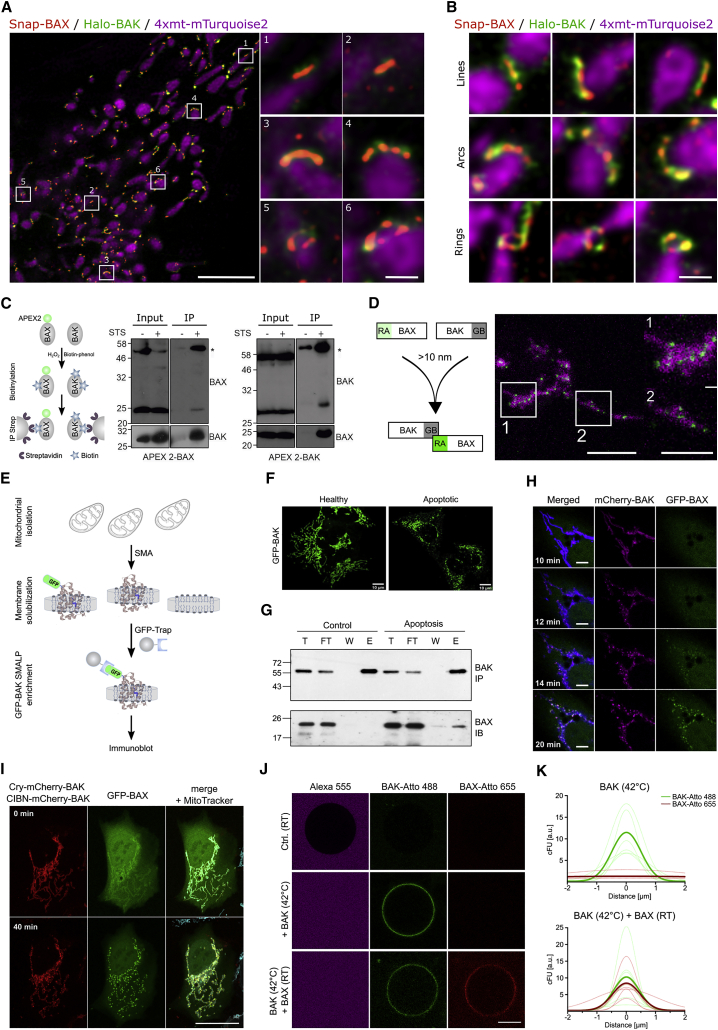
Recruitment of BAX by BAK and BAX-BAK co-localization (A) Live-cell STED super-resolution microscopy in BAX/BAK DKO U2OS cell transfected with Snap-BAX (red), Halo-BAK (green) and 4xmt-mTurquoise (magenta, confocal) to stain mitochondria after apoptosis induction. Zoomed images (right) correspond to crops of the overview image (left) as indicated by numbers. Scale bars, 5 μm and 500 nm for cropped images. (B) Gallery of BAX and BAK line, arc, and ring structures in apoptotic mitochondria by STED microscopy. Scale bars, 500 nm. Images in (A) and (B) are representative of three independent experiments. (C) (Left) Scheme of proximity-dependent labeling with APEX2 (see STAR Methods). (Central and right) Immunoblots of streptavidin immunoprecipitation of APEX2-BAX (central) or APEX2-BAK (right) from apoptotic HeLa cells expressing FLAG-APEX2-BAX or FLAG-APEX2-BAK. Immunoblots are representative of two independent experiments. Only regions of the gel with bands of interest are shown for clarity. (D) (Left) Principle of dimerization-dependent fluorescent protein (ddFP). RA only becomes fluorescence when it is in complex with GB. (Right) Representative confocal microscopy image of a BAX/BAK DKO U2OS cell transfected with GB-BAK, RA-BAX, and Mito-BFP (magenta) after apoptosis induction. Zoomed images correspond to cropped regions as indicated. The interaction between BAX and BAK (green dots) takes place in mitochondria upon apoptosis (negative control for collisions is not shown, it was tested in independent experiments). Scale bars, 10 μm; crop, 5 μm. Images are representative of two independent experiments. (E) Schematic representation of sample preparation for SMALPs pull-down assay (see STAR Methods). (F) Representative healthy (control) or apoptotic cells transfected with mEGFP-BAK (green) used in the assay. (G) Immunoblots of SMALPs immunoprecipitation of GFP-BAK and pull-down of endogenous BAX. Immunoblots show total input (T), flow through (FT), wash (W), and elution (E) fractions of the immunoprecipitation and are representative of three independent experiments. IB, immunoblot; IP, immunoprecipitation. Only regions of the gel with bands of interest are shown for clarity. (H) Representative confocal microscopy image of a BAX/BAK DKO U2OS cell transfected with mCherry-BAK (magenta), mEGFP-BAX (green), and Tomm20-BFP (blue) at indicated time points after apoptosis induction showing BAK foci appearing before BAX foci. Scale bars, 10 μm. Images are representative of three independent experiments. (I) Representative confocal microscopy image of optogenetic activation of BAK and recruitment of BAX to apoptotic mitochondria (MitoTrackerTM Deep Red FM, cyan). Scale bars, 20 μm. Images are representative of 4 independent experiments. (J) Representative images of BAK (green) activation followed by BAX (red) recruitment on a GUV (black hole). Alexa 555 (magenta) is used as a soluble dye to detect GUV permeabilization. Scale bars, 10 μm. Images are representative of 3 independent experiments. (K) Quantification of the binding of BAK-Atto 488 (green) and BAX-Atto 655 (red) to GUVs by radial profile corrected fluorescence intensity (cFU) measurements. Thin lines indicate individual measurements and thick lines correspond to the average of n = 7 individual GUVs from n = 3 independent experiments. See also Figure S7.

These results suggested that BAX and BAK may be part of the same high-order complexes. To address this question, we implemented dual-color super-resolution STED microscopy of Snap-BAX and Halo-BAK in combination with confocal microscopy of the mitochondrial network labeled with 4xmt-mTurquoise2 (Figure 6A). In living cells undergoing apoptosis, we identified individual lines, arcs, and rings formed by both proteins (Figures 6A, 6B, and S7A), revealing that BAX and BAK co-assemble into the same supra-molecular structures. Importantly, these results also provide direct visualization of the apoptotic pore formed by both BAX and BAK.

In agreement with this, the use of *in situ* proximity labeling with the engineered ascorbate peroxidase 2 (APEX2 [Lam et al., 2015]) confirmed that BAX was in direct vicinity of BAK and vice versa in the context of intact apoptotic foci, but not in healthy cells (Figure 6C). We further demonstrated the interaction between active BAX and BAK at individual apoptotic foci by dimerization-dependent fluorescent protein (ddFP) (Figure 6D), which only produces a fluorescent signal for co-expressed RA-BAX and GB-BAK proteins that are closer than 10 nm (Ding et al., 2015). Additional evidence of the association between BAX and BAK was obtained from co-immunoprecipitation (coIP) experiments using styrene maleic anhydride (SMA) co-polymers, which enable mild solubilization of mitochondria into lipid/protein nanoparticles of around 10 nm in diameter (Figure S7B) with the advantage of preserving the lipid environment (Lee et al., 2016). Following immuno-isolation of SMA lipid particles specifically containing GFP-BAK, we detected interaction with BAX exclusively under apoptotic conditions (Figures 6E–6G). Together, these results further support that BAX and BAK co-assemble into the same macromolecular complexes in apoptotic foci, whose properties depend on the ratio between BAX and BAK molecules.

The findings that BAK oligomerized faster and accelerated BAX assembly into the same supra-molecular complexes led us to speculate that BAK oligomers at mitochondria may act as seeding points for recruiting and activating cytosolic BAX. In agreement with this, we found that at early stages of apoptosis induction, mCherry-BAK clusters appeared before those of mEGFP-BAX at apoptotic foci (Figure 6H), independently of their expression levels. Additionally, we developed an optogenetic system for the light-controlled activation of BAK. Illumination with 405 nm light induced the oligomerization and redistribution into foci of CRY2-mCherry-BAK and CIBN-mCherry-BAK in BAX/BAK DKO U2OS cells also expressing mEGFP-BAX. Interestingly, light-induced BAK oligomerization caused the recruitment and accumulation of BAX at the same foci in absence of apoptotic stimuli, which led to apoptosis induction (Figure 6I). Further demonstration was provided by experiments in chemically defined minimal systems based on GUVs. Heat-activated BAK-488 bound to and permeabilized GUVs as expected. Remarkably, membrane-bound, active BAK-488 was able to recruit inactive, soluble BAX-655 to the GUVs in absence of any other components, as shown by the red fluorescence intensity on the GUV rim (Figures 6J and 6K). These results suggest that BAK accelerates the growth of BAX foci likely by direct recruitment and activation of cytosolic BAX.

### The interplay between BAX and BAK tunes the kinetics of mtDNA release and cGAS/STING-mediated inflammatory signaling

**Figure 7:**
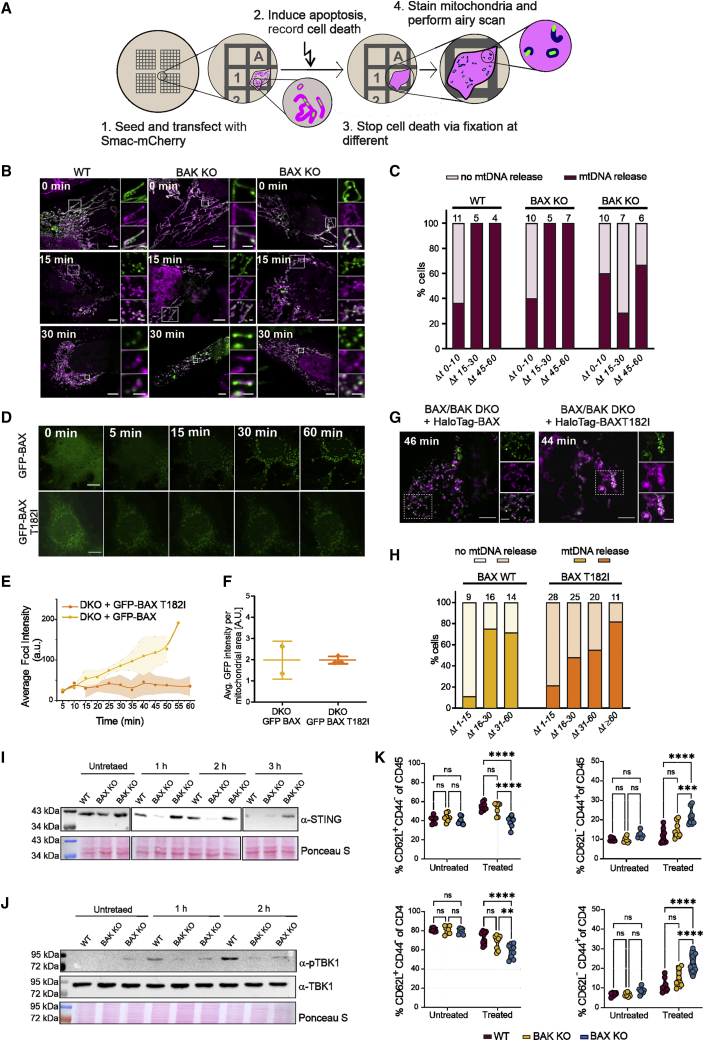
BAK pores enable faster mtDNA release and downstream inflammatory responses than BAX pores (A) Scheme of the experimental setup to investigate the kinetics of mtDNA release (green) after Smac(1-60)-mCherry (magenta) release by airy scan super-resolution microscopy. (B) Representative airy scan microscopy images of WT, BAK KO, and BAX KO U2OS cells transfected with Smac-(1-60)-mCherry, Tfam-GFP (green) and immunostained for Tomm20 (magenta) without induction of apoptosis (0 min) and 15 and 30 min after Smac(1-60)-mCherry release. Cropped panels on the right represent zoomed region of the white box in the main image. Scale bars, 5 μm and 1 μm for the main and zoomed images, respectively. (C) Percentage of cells with mtDNA release at different time points after Smac release. Numbers of analyzed cells (n) is on top of the bar graphs. (D) Representative photon-counting confocal images of BAX/BAK DKO U2OS cells transfected with mEGFP-BAX or mEGFP-BAX(T182I), after apoptosis induction. Time points are after Smac-mCherry release. Scale bars, 10 μm. (E) Average foci intensity at each time point for individual apoptotic cells expressing mEGFP-BAX (yellow, n = 2) or mEGFP-BAX(T182I) (orange, n = 3). Lines in the graph correspond to the average values from all measured cells and colored areas to data variability (mean ± SD). (F) Average mEGFP expression level detected by single-cell fluorescent intensity analysis per mitochondrial area in the individual cells (represented by individual dots in the plot) analyzed in (E). The error bars correspond to the SD from the different experiments. (G) Representative airy scan microscopy images of cells transfected with Smac-(1-60)-mCherry, HaloTag-BAX or HaloTag-BAX(T182I), Tfam-GFP (green) and immunostained for Tomm20 (magenta) at indicated time points after Smac release. Cropped panels on the right are zoomed region of the white box in the main image. Scale bars, 5 and 2 μm for the main and zoomed images, respectively. (H) Percentage of cells with mtDNA release at different time points after Smac release. Numbers of analyzed cells (n) is presented on top of the bar graphs. (I and J) STING degradation (I) and TBK1 phosphorylation (J) in WT, BAX KO, and BAK KO U2OS cells at 0 (untreated), 1, 2, and 3 h after apoptosis induction. Only regions of the gel with bands of interest are shown for clarity. (K) Changes of CD62L and CD44 expression in spleenocytes (CD45^+^, top row) and CD4^+^ T cells (bottom row) with SVEC cells either untreated or pre-treated with ABT-737, S63845 and QVD for 3 h. Two-way ANOVA and Sidak multiple comparison test was used for the statistical analyses. n = 8 spleen donors. ^∗∗^p < 0.01, ^∗∗∗^p < 0.001, ^∗∗∗∗^p < 0.0001. See also Figure S8.

The observation that BAK has faster kinetics of assembly and different cluster size compared with BAX raised the question whether this might have functional consequences during apoptosis. We reasoned that the different high-order assembly properties of BAX and BAK might have a stronger impact on the release of large macromolecules, such as mtDNA, from mitochondria. To test this hypothesis, we performed correlative live-cell confocal and fixed-cell airyscan super-resolution microscopy to follow the kinetics of mtDNA release with respect to the initiation of MOMP, measured as Smac-mCherry release, in single cells in the presence of either only endogenous BAX (BAK KO U2OS) or BAK (BAX KO U2OS), or both (WT U2SO) (Figures 7A and S8A). While in untreated cells mtDNA completely localized within mitochondria, we could detect mtDNA release after MOMP in all three cell lines, indicating that BAX and BAK are independently capable of forming large enough pores to meditate this process (Figure 7B). To our surprise, in cells expressing only BAK, all analyzed cells released mtDNA within 30 min after Smac-mCherry release, while in cells expressing only BAX, 25% of the cells still retained mtDNA within mitochondria even 60 min after Smac-mCherry release (Figure 7C). WT cells, containing both endogenous BAK and BAX, showed a similar phenotype to cells expressing only BAK. These results indicated that the kinetics of mtDNA release relative to Smac release was accelerated by BAK, in line with its increased assembly dynamics, and thus that the assembly rate of BAX and BAK is the main determinant for the kinetics of mtDNA release (Figures 5F and 5G).

In addition, using a BAX mutant that retains activity but presents reduced oligomerization, BAX(T182I) (Kuwana et al., 2020), which we validated at the single particle level in cells (Figures 7D–7F), we detected delayed mtDNA release compared with WT GFP-BAX (Figures 7G and 7H). These results hence provide a causative link between the assembly rate of BAX and BAK and the kinetics of mtDNA release independent of the genetic background. They also suggest a correlation between the number of BAX and BAK molecules at mitochondrial foci and the size of the apoptotic pore.

Since mtDNA release has been shown to induce the cGAS/STING pathway and to affect the inflammatory outcome of apoptosis (Rongvaux et al., 2014; White et al., 2014; Giampazolias et al., 2017), we compared the consequences of inducing MOMP solely by BAX, or BAK, or both, on inflammation. Under conditions of impaired caspase activity, we found that BAK-mediated MOMP in BAX KO SVEC cells led to faster STING degradation and TANK-binding kinase 1 (TBK1) phosphorylation, compared with WT SVEC cells or cells only expressing BAX (BAK KO SVEC cells), in line with an increased activation of the pathway (Figures 7I and 7J). Finally, we also evaluated the potential differences of BAX- and BAK-mediated MOMP on immune cells. To this aim, we co-cultured WT, BAK KO, and BAX KO SVEC cells, pre-treated with a combination of BH3 mimetics and a pan-caspase inhibitor, with primary mouse splenocytes. Remarkably, we detected a significant increase in the fraction of CD45^+^ hematopoietic cells showing hallmarks of T cell activation, i.e., loss of the L-selectin, CD62L, and increase in the cell surface protein CD44, that had been co-cultured with BAX KO SVEC cells. The CD4^+^ T helper cell population presented the highest differences (Figures 7K and S8B–S8D).

Together, these findings demonstrate that the coordinated assembly of BAX and BAK regulates the growth rate of the apoptotic pore and its permissiveness to large macromolecules, most notably mtDNA. We also uncover a key role of post-MOMP oligomerization kinetics of BAX and BAK in mtDNA release with functional consequences for the activation of the cGAS/STING pathway and of CD4^+^ T helper cells.

## Discussion

Here, we report a previously unrecognized function of BAX and BAK in timing the release of mtDNA and thereby modulating mtDNA-mediated inflammation in apoptosis. We demonstrate that the balance between BAX and BAK molecules defines the relative release kinetics of mitochondrial content, such as Smac and mtDNA, upon MOMP. The mechanistic basis for this function emerges from the distinct oligomerization dynamics of BAX and BAK and from their reciprocal regulation as they co-assemble into supra-molecular structures, which together determine the growth rate of the resulting apoptotic pore. This is important not only because it shows that the apoptotic pore size can be dynamically regulated but also because it affects downstream inflammatory signaling in apoptosis.

In this study, we provide direct structural evidence of BAK membrane pores. As previously shown for BAX, in apoptotic cells BAK assembles into heterogeneous structures that we classify as lines, arcs, and rings, with both arcs and rings being functionally able to perforate the membrane. These findings indicate a similar mechanism of membrane permeabilization for BAX and BAK based on lipidic pores, where the pore rim does not need to be fully covered by protein molecules to stabilize the open pore state. But in contrast to BAX, BAK oligomers and pores appear smaller and more homogeneous in size not only in cells but also in model membranes. This smaller size of BAK structures may be the reason why previous attempts failed to visualize BAK rings at apoptotic foci (Nasu et al., 2016).

We also show that the differences between BAX and BAK not only concern the size of the macromolecular complexes they form but also their assembly dynamics. Our quantitative analysis of individual apoptotic foci in dying cells demonstrates that BAK oligomers form faster and reach a stable average size in 5–10 min after MOMP. In contrast, BAX oligomers continue to grow almost linearly during apoptotic progression without an apparent size limit. Of note, these differences between BAX and BAK are not simply the result of different expression levels, molecular depletion or subcellular localization, as the mitochondria-resident BAX(S184V) mutant is expressed at similar levels to BAK but behaves similar to BAX WT. Together, our findings reveal underlying differences in the assembly of BAX and BAK and challenge the general assumption that these two proteins have fully overlapping mechanisms of action during apoptosis.

At the molecular level, the faster assembly and initial higher density of BAK clusters per membrane area observed could result from BAK presenting more effective intra-dimer interactions compared with BAX. In line with this hypothesis, recently, Iyer et al. showed a higher ability of BAK than BAX to activate other BAK and BAX molecules (Iyer et al., 2020). We cannot discard that other factors, including enhanced BAK accessibility to the BH3 domains of BAX and BAK (Iyer et al., 2020) or different association/dissociation kinetics with interacting partners in the cell, such as other BCL-2 proteins or VDAC2 (Ma et al., 2014; Cheng et al., 2003; Chin et al., 2018; van Delft et al., 2019), may also play a role.

Despite forming smaller structures, BAK oligomers in the cell can still reach hundreds of units and the resulting pores are big enough to release big macromolecules, such as mtDNA (Riley et al., 2018; McArthur et al., 2018; Figures 4 and 7). This raises the question about the role of persisting BAX oligomerization. It will be interesting to explore whether BAX cluster growth is a regulated process or just an uncontrolled consequence of its oligomerization and whether this has functional consequences beyond the regulation of BAK assembly shown here. In this regard, Adar et al. recently reported the presence of BAX “sponge” assemblies next to mitochondria at late stages of apoptosis (Ader et al., 2019) that may be the result of such large BAX oligomers reshaping and sequestrating outer membrane patches for membrane rupture.

Yet, unexpectedly, we found that the difference in the rate of assembly between BAX and BAK, and not their final pore size, is determinant for the timing of mtDNA release and, consequently, cGAS/STING pathway induction when caspase activity is perturbed. Another key discovery of our study is the reciprocal regulation of BAX and BAK during the growth of the apoptotic pore by co-assembly into the same supra-molecular line, arc, and ring-like structures. BAX slows down apoptotic cluster growth, yet it participates in and reshapes the architecture of BAK pores, which then reach bigger sizes at later stages of MOMP. In turn, BAK accelerates the kinetics of BAX foci assembly, likely by providing seeding points at the membrane. Although in the cellular context BAK is not essential for BAX activation (Chen et al., 2015; Ma et al., 2014; Chin et al., 2018), our experiments with optogenetic control in cells and with minimal systems in GUVs demonstrate direct activation of BAX by BAK. Importantly, the two-color super-resolution imaging provides a direct visualization of individual apoptotic pores containing both BAX and BAK molecules at mitochondria.

As a result of this functional interplay between BAX and BAK, their relative expression levels control not only apoptosis sensitivity, but also the dynamics of the orderly release of mitochondrial contents during MOMP, and thereby the activation of downstream pathways. We show that the timing of mtDNA release resulting from the differential assembly rate of BAX and BAK can impact the activation of cGAS/STING signaling in a cell-autonomous manner and promotes the expression of activation markers in bystander T helper cells in a paracrine fashion. A recent study reported that mtDNA triggered IFN production in a model of radiation therapy in breast cancer cells leading to immunogenic cell death and revealed that caspase activation after MOMP does not provide sufficient proteolytic activity to dampen IFN signaling in this context (McArthur and Kile, 2020; Yamazaki et al., 2020). Considering this, our findings suggest that the regulation of apoptotic pore growth by BAX and BAK upon MOMP enables tuning the timing and extent of caspase activation with respect to cGAS/STING signaling with an impact on inflammatory responses.

In summary, here, we show that BAX and BAK present distinct oligomerization properties during apoptosis and that they regulate each other. As a result, their co-assembly into the same supra-molecular structures modulates the relative kinetics of mitochondrial content release. This dynamic interplay between BAX and BAK provides an additional level of regulation for the growth of the apoptotic pore, which, as we directly visualized here, is formed by both BAX and BAK molecules. Our findings support a model in which BAK facilitates the fast recruitment and activation of BAX in the early stages of MOMP, while, once in the membrane, BAX sustains the slower accumulation of additional BAX molecules to growing pores. As a result, the co-assembly of BAX and BAK tunes the kinetics of mtDNA release relative to Smac, which impacts the downstream cGAS/STING signaling and bystander immune cells.

### Limitations of the study

Here, we show that BAX and BAK present distinct assembly properties that affect the relative timing of release of mitochondrial content. Yet, considering the overall similarity of BAX and BAK structures, the structural basis for these dynamic differences remains an open question, and it will require further structural investigation of the macromolecular complexes they form with atomic resolution. While our results with the oligomerization defective BAX mutant suggest a link between the supra-molecular assembly and permeabilization to larger content, like mtDNA, we could not find a clear-cut temporal evolution from lines, to arcs and to rings due to technical challenges. It thus remains to be determined whether the different structures detected for BAK and BAX are all relevant for MOMP and what makes the different shapes happen.

In addition, the use of overexpressed GFP-fused BAX or BAK constructs in the DKO and single KO cell lines to study the assembly of BAX/BAK on mitochondria could alter the extent of oligomerization. While overexpression of BAX and BAK is not very high because cells expressing large amounts of these proteins die, the use of genome editing to label endogenous BAX and BAK is certainly a route to address this issue. Along these lines, although we cannot exclude that the recruitment of BAX might be amplified by BAK overexpression, this does not change our conclusion about the ability of active BAK to recruit BAX.

Finally, regarding the initial stages of BAX and BAK assembly in cells at the moment of Smac release, we could only detect the first clusters, already with several tens of units, a few minutes later. The inability to visualize BAX and BAK oligomers preceding MOMP is common to other studies (Maes et al., 2017; Riley et al., 2018; McArthur et al., 2018) and could be due to technical limitations in detecting the fluorescence signal of fast-assembling small oligomers due to low contrast with the background. Although the question about the minimal number of BAX/BAK sufficient to initiate the apoptosis cascade remains open, it is now evident that the BAX and BAK higher-order oligomers characterized here are functionally relevant for mtDNA release in apoptosis (McArthur et al., 2018; Riley et al., 2018). Further methodological developments will hopefully allow tackling these relevant questions in the future.

## STAR★Methods

### Key resources table

**Table:** 

REAGENT or RESOURCE	SOURCE	IDENTIFIER
**Antibodies**		

Alpaca anti-GFP VHH sdAb AF647 (anti-GFP nanobody-AF647) 1:2000 dilution	Chromotec (home labeled)	Cat#gt-250
Rabbit polyclonal anti-BAX 1:1000 dilution	Cell Signaling Technology	Cat#2772; RRID: AB_10695870
Rabbit monoclonal anti-BAX (D2E11) 1:1000 dilution	Cell Signaling Technology	Cat#5023; RRID: AB_10557411
Mouse monoclonal anti-GFP 1:1000 dilution	ThermoFischer	Cat#MA5-15256; RRID: AB_10979281
Rabbit polyclonal anti-GAPDH 1:2500 dilution	abcam	Cat#ab9485; RRID: AB_307275
Goat polyclonal anti-Rabbit IgG-HRP 1:10000 dilution	Jackson Immuno Research	Cat#111-035-003; RRID: AB_2313567
Goat polyclonal anti-Mouse IgG-HRP 1:10000 dilution	Jackson Immuno Research	Cat# 115-035-003; RRID: AB_10015289
Camelid sdAb anti-GFP; Clones 1H1(FluoTag-Q anti-GFP Alexa Fluor 647)1:2000 dilution	NanoTag Biotechnologies	Cat#N0301
Goat polyclonal anti-Rabbit (AF633) 1:200 dilution	ThermoFisher	Cat#A-21070; RRID: AB_2535731
Rabbit monoclonal anti-Tom20 (D8T4N)1:100 dilution	Cell Signaling Technology	Cat#42406; RRID: AB_2687663
Mouse anti-Cytc 1:1000 dilution	BD Biosciences	Cat#556433; RRID: AB_396417
Mouse monoclonal anti-GAPDH (D4C6R)1:1000 dilution	Cell Signaling Technology	Cat#97166; RRID: AB_2756824
Rabbit anti-IMMT 1:1000 dilution	Proteintech	10179-AP; RRID: AB_2127193
Rabbit polyclonal anti-PARP 1:1000 dilution	Cell Signaling Technology	Cat#9542; RRID: AB_2160739
Rabbit monoclonal anti-pTBK1/NAK (Ser172) (D52C2)1:1000 dilution	Cell Signaling Technology	Cat#5483; RRID: AB_10693472
Rabbit monoclonal anti-TBK1/NAK (D1B4) 1:1000 dilution	Cell Signaling Technology	Cat#3504; RRID: AB_2255663
Rabbit monoclonal anti-STING (D2P2F) 1:1000 dilution	Cell Signaling Technology	Cat#13647; RRID: AB_2732796
PerCP/Cyanine5.5 Rat anti-mouse Ly-6C 1:200 dilution	Biolegend	Cat#128011; RRID: AB_1659242
Alexa Fluor 488 Rat anti-mouse/human CD44 1:800 dilution	Biolegend	Cat#103016; RRID: AB_493679
Alexa Fluor 647 Mouse anti-mouse NK-1.1 1:200 dilution	Biolegend	Cat#108719; RRID: AB_493186
Alexa Fluor 700 Rat anti-mouse CD4 1:100 dilution	Biolegend	Cat#100536; RRID: AB_493701
Super Bright 702 Rat monoclonal anti-CD45 (30-F11)1:200 dilution	ThermoFischer	Cat#67-0451-82; RRID: AB_2662424
Brilliant Violet 785 Rat anti-mouse CD62L 1:800 dilution	Biolegend	Cat#104440; RRID: AB_2629685
Brilliant Violet 650 Armenian Hamster anti-mouse CD80 1:400 dilution	Biolegend	Cat#104732; RRID: AB_2686972
Brilliant Violet 421 Rat anti-mouse/human CD11b 1:400 dilution	Biolegend	Cat#101251; RRID: AB_2562904
Brilliant Violet 510 Rat anti-mouse CD8a 1:200 dilution	Biolegend	Cat#100752; RRID: AB_2563057
Super Bright 600 Rat monoclonal anti-CD45R (B220) (RA3-6B2) 1:200 dilution	ThermoFischer	Cat#63-0452-82; RRID: AB_2637457
PE-Cy7 Hamster anti-mouse CD11c 1:400 dilution	BD Biosciences	Cat#561022; RRID: AB_647251
PE Rat anti-mouse Ly-6G 1:800 dilution	Biolegend	Cat#127607; RRID: AB_1186104
PE/Dazzle 594 Rat anti-mouse I-A/I-E 1:800 dilution	Biolegend	Cat#107647; RRID: AB_2565978
Rat anti-mouse CD16/CD32 1:50 dilution	BD Biosciences	Cat#553142; RRID: AB_394656

**Bacterial and virus strains**		

BL21 (DE3) RIPL	Stratagene (available via Agilent)	Cat#230280

**Chemicals, peptides, and recombinant proteins**		

Egg PC	OttoNordwald (Avanti)	Cat#840051C
Cardiolipin	Sigma	Cat#C0563
DiI stain	ThermoFischer	Cat#D3911
S63845	Hölzel	Cat#HY-100741
ABT-737	Hölzel	Cat#HY-50907
Q-VD-OPh	Hölzel	Cat#HY-12305g
Staurosporine	LC laboratories (USA)	Cat#S3900
GLUCOSE OXIDASE TYPE VII	Sigma	Cat#G2133-10KU
Cysteamine-Hydrochlorid (MEA)	Sigma	Cat#95294-1L
Catalase	Merck	Cat#219261-100KU
SNAP-cell 647SiR	NEB	Cat#S9102S
Janelia Fluor® 549 HaloTag® Ligand	Promega	Cat#GA1110
Live Cell Imaging Solution	ThermoFischer	Cat#A14291DJ
Pierce™ Streptavidin Magnetic Beads	ThermoFischer	Cat#88817
GFP Trap agarose beads	Chromotek	Cat#X336.1
XIRAN® (SMA2:1)	Polyscope	Cat#SZ30010

**Critical commercial assays**		

Live/Dead fixable Near-IR Cell Stain Kit	ThermoFischer	Cat#L10119

**Deposited data**		

Raw western blot and microscopy data	Mendeley	doi: 10.17632/2gwcgpvwx5.1

**Experimental models: Cell lines**		

Human bone osteosarcoma cells: U2OS	Gift from S. Tait, Glasgow	https://doi.org/10.15252/embj.201899238
Human bone osteosarcoma cells: U2OS BAX ^-^/^-^ (U2OS BAX KO)	Gift from S. Tait, Glasgow	https://doi.org/10.15252/embj.201899238
Human bone osteosarcoma cells: U2OS BAK ^-^/^-^(U2OS BAK KO)	Gift from S. Tait, Glasgow	https://doi.org/10.15252/embj.201899238
Human bone osteosarcoma cells: U2OS BAX ^-^/^-^/BAK ^-^/^-^(U2OS BAX/BAK DKO)	Gift from S. Tait, Glasgow	https://doi.org/10.15252/embj.201899238
Human bone osteosarcoma cells: U2OS NUP96-mEGFP	Available from CLS cell lines service GmbH	Cat#300174
Murine endothelial cells: SVEC 4–10	Gift from S. Tait, Glasgow	https://doi.org/10.15252/embj.201899238
Murine endothelial cells: SVEC 4–10 BAX -/-(SVEC BAX KO)	Gift from S. Tait, Glasgow	https://doi.org/10.15252/embj.201899238
Murine endothelial cells: SVEC 4–10 BAK -/-(SVEC BAK KO)	Gift from S. Tait, Glasgow	https://doi.org/10.15252/embj.201899238
Human colon cancer cells: HCT116 BAX ^-^/^-^/BAK ^-^/^-^(HCT116 BAX/BAK DKO)	Gift from K. Schulze-Osthoff, Tübingen	N/A
Spleenocytes extracted from female mice (C57BL/6N)	Available from The Jackson Laboratory	Cat#005304

**Recombinant DNA**		

pcDNA3_Smac1-60_mCherry	Gift from S. Tait, Glasgow	https://doi.org/10.15252/embj.201899238
mTagBFP2-TOMM20-N-10	Addgene	Cat#55328
pEGFP-A206K_BAX_C1 (mEGFP-BAX)	This manuscript	N/A
pEGFP-A206K_BAX(S184V) C1 (mEGFP-BAX(S184V))	This manuscript	N/A
pEGFP-A206K_BAX(T182I) C1 (mEGFP-BAX(T182I))	This manuscript	N/A
pEGFP_A206K_BAK_C1 (mEGFP-BAK)	This manuscript	N/A
pTYB21_mBAK-L192K, F193S, Y196D, C154S	This manuscript	N/A
pTYB1_hBAX-S4C,C62S, C126S	Previous study	https://doi.org/10.1038/ncomms9042
pTYB1 Bid WT	Previous study	https://doi.org/10.1038/ncomms9042
pEGFP-Tfam-GFP	This manuscript	N/A
pCRY2(1-531)-mCherry-BAK	Addgene	Cat#117238
pcDNA-CIBN-mCherry-BAK	This manuscript	N/A
pEGFP-N1-4xmt-mTurquoise2	Addgene	Cat#98819
pAcGFP-C1-SnapF-BAX	This manuscript	N/A
pAcGFP-C1-Halo7-BAK	This manuscript	N/A
pEGFP-Flag-APEX2-BAX	This manuscript	N/A
pEGFP-Flag-APEX2-BAK	This manuscript	N/A
pAcGFP-C1-RA-BAX	This manuscript	N/A
pAcGFP-C1-GB-BAK	This manuscript	N/A

**Software and algorithms**		

Stoichiometry Analysis Software (SAS)	https://doi.org/10.1021/acs.jpclett.1c03835	https://github.com/jdanial/SAS
GUVdetector software	https://doi.org/10.1093/bioinformatics/btu102	http://garcia-saez.cecad-labs.uni-koeln.de/GUV-software.864.0.html
ImageJ/FIJI	doi: 10.1038/nmeth.2019	https://imagej.net/software/fiji/
ThunderStorm	DOI: 10.1093/bioinformatics/btu202	https://code.google.com/p/thunder-storm/
Huygens Professional	Scientific Volume Imaging, Netherlands	http://svi.nl
Radial Profile Plot Plugin (ImageJ)	https://imagej.nih.gov/ij/plugins/radial-profile.html	N/A
GraphPad Prism 5.0	GraphPad	https://www.graphpad.com/scientific-software/prism/
Origin	OriginLab Corporation	https://www.originlab.com/
Python	https://www.python.org/	N/A

### Resource availability

#### Lead contact

Further information and requests for resources and reagents should be directed to and will be fulfilled by the lead contact, Ana J. García Sáez (ana.garcia@uni-koeln.de).

#### Materials availability

Plasmids generated in this study are available on request to the lead contact.

### Experimental model and subject details

#### Cell culture

WT, single BAX KO, single BAK KO, double BAX/BAK DKO and mEGFP-tagged NUP96 human osteosarcoma U2OS, WT, single BAX KO and BAK KO SVEC 4–10 murine endothelial and BAX/BAK DKO human colon carcinoma HCT116 cell lines were cultured at 37 °C and 5% CO_2_ in DMEM (McCoy's 5A for HCT116) supplemented with 10% FBS and 1% penicillin/streptomycin (Invitrogen, Germany). BAX/BAK DKO cell lines ensured that no endogenous BAX and BAK was contributing to foci formation. Cells were transfected at 70–80% confluence. All cell lines used in this study were subjected to regular mycoplasma testing.

#### Isolation of spleenocytes

Spleenocytes were collected from 9-13 week old female mice (C57BL/6N). For preparation of single cell suspension, the spleens were minced through 40 µm cellstrainer using a plunger and PBS for flushing. The single cell suspension was then centrifuged at 300 x g for 5 minutes, followed by supernatant removal and treatment with 1 mL ACK buffer (A1049201, ThermoFisher) for 2 minutes at room temperature. The treatment was stopped by addition of 20 mL PBS, centrifugation for 5 minutes at 300 x g and resuspension of the single cell solution in cell media.

### Method details

#### Plasmids and antibodies

The plasmids and antibodies used in this study are listed in the key resources table.

#### Protein purification and labelling

Full-length mouse Bid and single-cysteine, full-length human BAX mutant (S4C, C62S and C126S), were expressed in *Escherichia coli* and purified as described elsewhere (Desagher et al., 1999; Bleicken et al., 2010). Briefly, BAX and Bid were expressed in BL21 (DE3) RIPL cells from a pTYB1 and a pET15b expression vector, respectively, and isolated using chitin affinity chromatography followed by ion-exchange chromatography (BAX) and IMAC affinity chromatography (BID). Caspase-8 was used to cleave Bid (cBid). Protein quality was checked by SDS–polyacrylamide gel electrophoresis. Mouse BAK carrying a modified, more hydrophilic C-terminus (L192K, F193S, Y196D) (Leshchiner et al., 2013) and mutated to single-cysteine (C154S), was transformed into BL21 (DE3) RIPL cells (Stratagene, La Jolla, CA). Cultures were grown at 37°C in LB medium containing ampicillin (100 μg/ml) and chloramphenicol (35 μg/ml). Gene expression was induced by 1 mM IPTG (isopropyl-1-thio-β-D-galactopyranoside) at OD (600 nm) of 0.7. The temperature was lowered to 20°C, cells were harvested after an additional 16 h, frozen in liquid nitrogen and stored at -20°C. For purification, cells were resuspended in buffer A (1 M NaCl, 20 mM Tris, pH 8), ruptured by French press and separated by centrifugation. The BAK-containing supernatant was purified by incubation with chitin beads and treated for intein cleavage as described by the manufacturer (NEB) and further purified by size exclusion chromatography. Protein purity was analyzed by SDS-PAGE, LC-MS and western blot. For protein labelling, Atto 488 (Attotec, Siegen, Germany) dye was covalently attached to the single cysteine of BAX or BAK as described by the manufacturer. Excess of the label was removed by size exclusion chromatography. The activity of the unlabeled protein was tested by calcein assay as performed in Bleicken et al. (2013b). The activity of labelled proteins was controlled by giant unilamellar vesicle membrane permeabilization assay (Hermann et al., 2014). Labelling efficiency was calculated to be 75-82% for BAK and 84% for BAX by comparing protein and label concentrations with Bradford and spectrophotometer measurements.

#### Western blotting

Cells were lysed in RIPA buffer (150 mM NaCl, 0.5% sodium deoxycholate, 1% NP-40, 0.1% SDS, 50 mM Tris, pH 8.0) with protease and phosphatase inhibitors and pre-cleared. Protein concentration was determined by Bradford protein assay (Bio-Rad) according to the manufacturers protocol. Equal amounts of protein (30-100 μg, depending on the experiment) were loaded on a 4-12% Tris-Bis gel (Thermo Scientific) and transferred onto PVDF or nitrocellulose membrane using the Turboblot (BioRad). Blots were blocked with 5% milk in TBST and incubated overnight at 4 °C with the primary antibody (see key resources table), probed with secondary antibodies (see key resources table) and developed using ECL (BioTool).

For separation of mitochondria and cytosolic fractions, cells lysed in permeabilization buffer (20 mM HEPES/KOH pH7.5, 100 mM sucrose, 2.5 mM MgCl2, 100 mM KCl, freshly added 0.025% (w/v) digitonin and protease inhibitor cocktail in PBS) for 10 min on ice and total cellular membranes were pelleted by centrifugation at 15.000 x g for 10 min at 4°C. After removing the supernatant (cytosolic fraction), the membranes were solubilized using RIPA buffer as described above.

#### Supported lipid bilayers (SLBs)

All lipids were purchased from Avanti Polar Lipids. Egg phosphatidylcholine (EPC) and cardiolipin (CL) were mixed in an 8:2 ratio and dissolved in chloroform. To obtain proteoliposomes, large unilamellar vesicles (LUVs) of 100 nm were prepared as described elsewhere (Subburaj et al., 2015). Briefly, lipids were resuspended in buffer (150mM NaCl, 10mM HEPES/KOH, pH7.4) to a final concentration of 0.6 mg/ml and passed through five cycles of freezing and thawing after which they were manually extruded through a polycarbonate membrane of defined pore size (100 nm) using glass syringes. For stoichiometry measurements, LUVs were incubated with 1, 5 or 10 nM BAX-488 or BAK-488 activated by heat (43°C) or with 1 nM BAK-488 activated by 2 nM cBid. After 1 hour incubation time, BAX/BAK-containing proteoliposomes were diluted to a 1:2, 1:5 or 1:10 ratio (for 1, 5 and 10 nM protein concentration, respectively) with LUVs to be in the single-molecule regime. The resulting solution was immediately used to create SLBs on piranha-cleaned glass slides (0.13–0.16-mm thickness, Menzel; Figure 3H) (Subburaj et al., 2015). In control experiments, proteins were added after SLB formation and incubated for 30 min. Unbound proteins and non-fused vesicles were removed by careful washing with buffer and the SLBs were immediately imaged. For AFM measurements, LUVs were incubated with 200 nM heat-activated BAK for 1 hour incubation time at 43°C. Liposomes (for control measurements) or proteo-liposomes were immediately used to create SLBs on freshly-cleaved mica, like in Unsay et al. (2017). Unbound proteins and non-fused vesicles were removed by careful washing with buffer and the SLBs were immediately imaged.

#### GUV permeabilization assay

GUVs were produced by electro-formation and the experiments were done as described in Bleicken et al. (2013b). Briefly, 2.5 μl of a 2 mg/ml EPC:CL 80:20 lipid mixture solution, doped with the lipophilic membrane dye DiI (Thermo Fisher) and dissolved in chloroform, were spread on each platinum electrode of an electro-formation chamber and immersed in 300 mM sucrose. Electro- formation proceeded for 2 h at 10 Hz, followed by 30 min at 2 Hz. 50 μl of the GUVs suspension was added to a solution of PBS buffer mixed with the appropriate concentrations of the proteins of interest in Lab-Tek 8-well chamber slides (NUNC) to a final volume of 200 μl. To measure membrane permeabilization in steady-state conditions, a Cyt*c*488/APC mixture solution was added after 1-hour incubation with cBid-activated BAK at reported concentrations. Images were collected 30 min later. The percentage of GUVs internalizing the fluorescent probes was determined by the *GUVdetector* software described in Hermann et al. (2014).

#### Atomic force microscopy

SLBs were imaged using a JPK NanoWizard II system (JPK Instruments, Berlin, Germany) mounted on an Axiovert 200 Inverted Microscope (Carl Zeiss). Intermittent contact (IC or tapping) mode images were taken using V-shaped silicon nitride cantilevers with a typical spring constant of 0.09 N/m (SNL-10 Bruker). The cantilever oscillation was tuned to a frequency between 3 and 10 kHz, and the amplitude was set between 0.2 and 1 V. The amplitude was varied during the experiment to minimize the force of the tip on the bilayer. The scan rate was set between 0.5 and 1 Hz. The height, deflection and phase-shift signals were collected, simultaneously, in both trace and retrace directions.

Roughness was measured as the average peak-to-peak distance in a cross-section of a bilayer. Images were processed by the JPK processing software, applying a smoothing function. Bilayer thickness was measured based on the height profiles from the mica (membrane defects or pores) to the membrane bulk. The height of the structures around the pores was measured based on the height profile from the membrane bulk.

#### TIRF microscopy

All experiments were performed using a modified Zeiss Axiovert 200M epifluorescence microscope using a 488 laser (Ichrome MLE-LFA multi-laser, Toptica) equipped with an α Plan-Fluor 100x/1.46 oil objective (Zeiss), a Laser-TIRF 3 Imaging System (Zeiss) and an EM-CCD camera (iXon 897, Andor). Sample areas of 150x150 or 200 x 200 pixels (pixel size 100 nm) were illuminated for 35 ms with a delay time between frames of 25 ms (number of frames 1200) with an intensity of ∼ 0.1 kW/cm^2^.

#### Stoichiometry determination

The images acquired by TIRF microscopy were used for the stoichiometry analysis based on the fluorescence intensity of the particles (i.e. brightness analysis) using an in-house develoed Stoichiometry analysis software (SAS) (Danial et al., 2022) implemented in Matlab which is available on github (https://github.com/jdanial/SAS).

Bright spots were automatically detected using an implementation of the Difference of Gaussians method (skimage.feature.blob dog http://scikit-image.org/docs/dev/api/skimage.feature.html) and thresholding. Selected particles were defined by a region of interest (ROI) of defined pixel size (3 pixels in radius) and fitted to two-dimensional (2D) Gaussians. Background subtraction was performed by defining a ROI around the particlés ROI having a larger pixel size (4 pixels in radius). This algorithm provided the brightness value for each spot. Localized particles were filtered based on the distance between two ROIs, to avoid overlapping ROIs, and on the presence of multiple particles in the same ROI. To this aim, we implemented a method that allows detecting two or more adjacent particles in one ROI by measuring the full width at half maximum (σ) of the fitted 2D Gaussian curves (see Jenner et al., 2020; STAR Methods section “Stoichiometry analysis by ratiometric approach”). Stoichiometry counting was performed using the brightness analysis method as previously described (Subburaj et al., 2015). The obtained brightness values were plotted as a probability density function (Pdf) or as a Gaussian distribution. We calibrated the fluorescence signal of the TIRF microscope before each experiment to avoid artefacts due to small changes in the optical setup. Monomeric BAK-488 particles were obtained by adding BAK-488 directly on an SLB (in which case no oligomerization occurred due to unspecific interactions of BAK with the glass support, Figure S4) and selected by photobleaching analysis (Subburaj et al., 2015; Ulbrich and Isacoff, 2007) after smoothing of the signal with a median filter. Correction for partial labelling efficiency was performed as previously described (Subburaj et al., 2015). Only proteins having >75% labelling efficiency were considered for experiments and data collection. The graphs with the percentage of occurrence refer to the average percentage of occurrence of fluorophore units per particle obtained from at least two different experiments (minimum 1500 particles per experiment from proteoliposomes and 600 particles per experiment of protein directly added to the SLB), and the error bars correspond to the standard deviation.

#### Analysis of BAX/BAK assembly kinetics

The assembly kinetics of BAX and BAK were investigated using photon counting confocal microscopy. Cells were transfected with 50 ng of pEGFP-A206K-BAX-C1 or pEGFP-A206K-BAK-C1 and 100 ng pcDNA3_Smac1-60_mCherry (see key resources table), using 0.5 μl Lipofectamine 2000 per well in a glass-bottom 8-well μ-slide (IBIDI) 12 h before confocal imaging. Apoptosis was induced with 1 μM of staurosporine (Sigma, Germany) or 1 μM ABT-737, 1 μM S63845 (MedChemExpress) and 10 μM qVD-OPh (APEXBIO). Confocal microscopy was performed on an LSM 710 (Carl Zeiss Microimaging) inverted microscope equipped with a Plan-Apochromat 63x/1.4 oil immersion objective (Zeiss) and an Argon LASER (LGN 3001, Lasos Lasertechnik). Pinhole size was adjusted to yield a depth of field of 1 Airy unit (∼800 nm for GFP emission). Before imaging, the growth medium was changed to 200 μl of phenol-red free DMEM and cells were maintained at 37 °C and 5% CO_2_ during imaging. Cells were imaged by selecting a region of interest (ROI) and finding the focal plane of the cell using the ‘fast’ scanning mode of the microscope with a greatly reduced laser power. Then, z-stacks of 8 images were collected with an interval of 300 nm, which was set based on the point-spread function analysis of fluorescent beads as the maximum interval required to record at least 80% of the maximum possible intensity from any point source. Images of 44.98 x 44.98 μm (512x512 pixel) were acquired with a laser pixel dwell of 3.15 μsec and an averaging of 2 images. Single emission photons were detected on an Avalanche Photodiode (APD) detector. Kinetics analysis of mEGFP-BAX or mEGFP-BAK oligomerization was performed by photon-counting fluorescence microscopy acquiring z-stacks every 5 minutes (for a time series of 60 minutes) after MOMP. MOMP was indicated by the release of Smac-1-60-mCherry (a truncated version of Smac targeting the intermembrane space, from now on Smac-mCherry) from mitochondria to the cytosol. We verified that using these imaging parameters, we did not have significant bleaching of our samples due to laser exposure.

#### Stoichiometry analysis by ratiometric approach

Single-particle detection and brightness analysis were performed on maximum intensity z-projections of individual time points (Figures S5A and S5B), using an in-house python algorithm. Selected particles were defined by a region of interest (ROI) of defined pixel size (2 pixels in radius) and fitted to two-dimensional (2D) Gaussians. Background subtraction was performed by defining a ROI around the particle's ROI having a larger pixel size (3 pixels in radius). Localized particles were filtered based on the width of the 2D Gaussian, which is indicative of the presence of multiple particles in the same ROI. Specifically, we implemented a method that allows detecting two or more adjacent particles in one ROI by measuring the full width at half maximum (σ) of the fitted 2D Gaussian curves. After plotting σ values of all ROIs as a normal distribution, those ROIs whose σ fell out the 95^th^ percentile of the distribution were excluded, as the bigger σ value is likely indicative of the presence of more particles in these ROIs (Figures S5C and S5D). Importantly, particles whose intensity was maximum in the outer frames were also discarded, as intensity information could be lost (Figure S5A). The stoichiometry (or molecularity) of mEGFP-BAX or mEGFP-BAK foci M_p_ were quantified by ratiometric comparison of their fluorescence intensity I_p_ to the fluorescence intensity of a standard I_s_, of known stoichiometry M_s_, tagged with the same fluorescent dye as the protein of interest, according to the formula:

$$M_{p}=I_{p}\;M_{s}/I_{s}$$

As an internal standard, we used the 32-mer mEGFP-tagged nuclear pore complex protein Nup96, endogenously expressed in the same cell line (Thevathasan et al., 2019). We additionally confirmed the validity of this standard by ratiometric comparison of the fluorescent intensity of Nup96-mEGFP complexes with the one of recombinant mEGFP-ferritin, which assembles as a 24-mer (Liße et al., 2017; Figures S5E–S5G). For each cell, the standard fluorescence intensity i_s_ was obtained as the mean value of the Gaussian-fitted fluorescence intensity distribution of Nup96-mEGFP oligomers within the cell. I_s_ represents the average value of i_s_ from at least 5 different imaged cells, measured on the same day of the experiment. For a given cell, at each time point, the values in molecular units for detected mEGFP-BAX or mEGFP-BAK foci were plotted as a probability density function or as cumulative counts. Exponential decay fitting of the cumulative distribution provided the average molecularity of mEGFP-BAX or mEGFP-BAK foci of individual cells at each time point.

#### Measurement of protein expression level

To determine the concentration of BAX and BAK in cells, we measured the expression levels of endogenous BAX and BAK or mEGFP-BAX and mEGFP-BAK in cell lysates. We used WT cells, and single BAX^-/-^, BAK^-/-^ or double BAX^-/-^ BAK^-/-^ cells transfected with mEGFP-BAX and mEGFP-BAK (see key resources table) for cell lysate production, after sorting for fluorescent cells by flow cytometry, and analysed them via Western Blotting. To measure the expression level in single living cells, the GFP intensity of each mEGFP-BAX/BAK transfected cell was imaged by confocal microscopy and measured using an in-house algorithm. The GFP intensity value of a cell was normalized to the area of its mitochondrial network, detected by measuring the number of pixels with Smac-mCherry signal before apoptosis induction. This ratio represents the expression level of mEGFP-BAX/BAK in each cell.

#### SMLM and Correlative fluorescence/SMLM imaging

For SMLM experiments, cells were transfected with 300 ng pEGFP-A206K-BAK (see key resources table) plasmid 16 h before the experiment. Apoptosis was induced by incubating the cells with 1 μM STS for 3 h. To determine the optimal time point for cell fixation after apoptosis induction, mEGFP-BAK transfected cells undergoing apoptosis were stained with TMRE (Life Technologies, Germany) to monitor mitochondrial depolarization. The loss of TMRE staining was analysed by flow cytometry and occurred for 50% of all mEGFP-BAK positive cells 3 hours after apoptosis induction. For correlative fluorescence/SMLM imaging, HCT116 or U2OS cells were seeded on μ-Dish^35 mm, high^ or μ-Dish^35 mm, high^ Grid-500 Glass Bottom chamber (IBIDI) . Apoptosis was induced with 1 μM ABT-737, 1 μM S63845 and 10 μM qVD-OPh. Foci formation of mEGFP-BAK was visualized in selected cells by acquiring images every 2 min using confocal microscopy performed on an LSM 710 ConfoCor3 microscope (Carl Zeiss) equipped with a Zeiss C-Apochromat 40x/1.2 water immersion objective. At individual time points after first foci appearance, cells were fixed in 4% paraformaldehyde in DMEM for 10 min, washed twice with PBS and incubated in quenching buffer (50 mM NH_4_Cl in PBS) for 15 min. Cells were permeablized with 0.25% Triton-X in PBS for 8 min, washed three times for 5 min with PBS and incubated in blocking buffer (1% BSA in PBS) for 45 min. Immunostaining was performed using a FluoTag-Q anti-GFP Alexa Fluor 647 nanobody (see key resources table) in 1:2000 dilution in blocking buffer for 1.5 h followed by washing three times for 5 min with PBS. For the SMLM imaging, 1.5 ml blinking buffer (50 mM Tris/HCl pH 8, 10 mM NaCl, 10% (v/w) glucose, 35 mM cysteamine (MEA), 0.5 mg/mL glucose oxidase, 40 g/ml catalase) was added. Image acquisition was performed on an Axiovert 200 Inverted Microscope (Carl Zeiss) equipped with a 100 mW 647 nm Coherent Obis diode laser and an α Plan-Fluor 100x/1.46 oil objective. Fluorescence emission was filtered using a 700/75 nm bandpass filter and imaged on an EM-CCD camera (iXon 897, Andor). Blinking of individual fluorophores was controlled by manually tuning the power of a 405 nm laser (iChrome MLE, Toptica photonics) to achieve a constant density of fluorescent molecules throughout the acquisition period. An exposure time of 30 ms was used and 70,000-100,000 frames recorded. Frame rate was 40 ms. Imaging laser intensity at 640 nm was 2.5 kW/cm^2^, and the 405-nm activation laser intensity was manually adjusted to keep a constant number of localizations per frame. The field of view varied depending on cell size or area of interest and pixel size was 100 nm. Focus was maintained to +/- 20 nm in the lateral position with the aid of a home-built autofocusing system using a back-reflected IR diode laser (LDM850,Thorlabs, 850 nm) with a Lateral effect position Sensor (PDQ80A, Thorlabs) and a piezo-stage interfaced through a PID circuit. Acquired images were analyzed using thunderSTORM (Ovesný et al., 2014). Individual fluorophore localizations were detected using a Difference of Gaussians implementation and fitted to Gaussian profiles using the Least Square method. For reconstruction of a super-resolved image the uncertainty in the fitting procedure combined with the sigma of the fitted Gaussians were used to improve the image quality and discard false localizations. Localizations with uncertainties above 20 nm were discarded.

#### Live cell STED microscopy

BAX/BAK DKO U2OS cells were seeded in 8-well glass bottom dishes (ibidi GmbH, Martinsried, Germany) and transfected with 50 ng pAcGFP-C1-SnapF-BAX (Snap-BAX), 50 ng pAcGFP-C1-Halo7-BAK (Halo-BAK) and 50 ng pEGFP-N1-4xmt-mTurquoise2 (mitoTruquoise, see key resources table) 16 hours prior to imaging. Cells were stained with 1 μM SNAP-Cell SiR (New England Biolabs) and 300 nM Janelia Fluor® 549 HaloTag® Ligand (Promega Corporation, 2800 Woods Hollow Road, Madison, WI 53711 USA) in DMEM for 20 min at 37 °C. After labeling, the cells were washed twice with DMEM and incubated for 15–30 min to remove unbound dye. Prior to imaging, the culture medium was changed to Live Cell Imaging Solution (Thermo Fisher Scientific) containing 1 μM ABT737, 1 μM S63845 and 10 μM and Q-VD-OPh (MedChemExpress) to induce apoptosis. Live cell STED microscopy was performed on a confocal laser scanning STED microscope (TCS SP8 STED 3x, Leica, Wetzlar) equipped with a HL PL APO 100x/1.40 Oil STED (white) objective (Leica, Wetzlar). Imaging was performed line sequentially with a pinhole size of 1.0 Airy unit and a pixel size of 23 nm. SNAP-Cell SiR was excited at 640 nm and Janelia Fluor® 549 at 561 nm. Both dyes were depleted at 775 nm wavelength. For triple color images, a confocal image of mitoTurquoise was acquired in addition, using the line sequential mode with an excitation wavelength of 458 nm. The fluorescence signal was detected using Hybrid detectors with a gating of 0.5-8 ns, a pixel dwell-time of 2.44 μs and 8 times line averaging. Images have been adjusted for brightness and contrast and processed by one smooth step using Fiji/ImageJ. Images were deconvolved with Huygens Professional version 19.04 (Scientific Volume Imaging, The Netherlands, http://svi.nl).

#### Proximity-dependent labeling with APEX2

Proximity-dependent labeling with APEX2 was done as described in Lam et al. (2015). In short, the APEX2 enzyme attached to BAX (or BAK) creates a free radical from Biotin-Phenol (BPh) in presence of H_2_O_2_ that reacts with neighboring (∼10 nm) proteins. After proximity-dependent biotinylation, cell lysates are used for immunoprecipitation with streptavidin (Figure 6C). HeLa cells were transfected with Flag-APEX2-BAX or -BAK (see key resources table) for 16h and incubated 3 h with 1 μM staurosporine at 37°C, 5% CO2 to induce apoptosis. Next, media was changed to DMEM with 500 μM biotin-phenol and cells were incubated for 30 min at 37°C, 5% CO2. Hydrogen peroxide (H2O2) was then added to a final concentration of 1 mM for 1 min at RT to initiate the biotinylation reaction. After quenching the biotinylation reaction, cells were lysed using RIPA buffer. A fraction of the whole cell lysate was stored (input) and equal amounts of whole cell lysates were incubated with 200 μl streptavidin-coated magnetic beads rotating for 1 h at 4°C. Beads were collected using a magnetic rack and were exhaustively washed on ice with RIPA buffer, 1 M potassium chloride, 0.1 M sodium carbonate, 2 M urea in 10 mM Tris-HCl pH 8.0, RIPA buffer and three times with 20 mM ammonium bicarbonate, pH 8.0, sequentially. After washing, the beads were boiled with SDS-PAGE loading buffer supplemented with 2 mM biotin for 5 min at 95°C. The supernatant fractions containing biotinylated proteins were loaded onto a polyacrylamide gel for detection of BAX (anti-BAX) and BAK (anti-BAK, see key resources table) by Western Blot.

#### Dimerization-dependent fluorescent protein

Dimerization-dependent fluorescent protein (ddFP) was done as described in Ding et al. (2015). HeLa cells were seeded in 8-well chambers (Ibidi) and transfected with RA-BAX, GB-BAK and mitoBFP (see key resources table). 1 μM STS was added in DMEM without phenol red media and cells were imaged after 3 h of apoptosis induction. Images were acquired using the water objective of the confocal microscope equipped with incubator at 37°C and 5% CO2.

#### Immunoprecipitation of GFP-BAK-containing SMALPs

SMA lipid particles (SMALPs) were generated from isolated mitochondria containing mEGFP-BAK and subjected to immuno-precipitation and immunoblotted with BAX and BAK antibodies (Figure 6E). For this, BAK KO U2OS cells transfected with mEGFP-BAK (see key resources table) were treated with 1 μM ABT737 and 1 μM S63845 to induce apoptosis and harvested 45 min post treatment. Apoptotic and control (untreated) samples were mechanically lysed using a dounce homogenizer and centrifuged at 600 x g to remove non-lysed cells and nuclei. Mitochondria were pelleted from the supernatant by centrifugation at 10,300 x g. Mitochondrial pellets were washed twice in isolation buffer (5 mM Tris/HCl pH 7.4, 250 mM sucrose, and 2 mM EDTA) and once with Tris buffer (50 mM Tris/HCl pH8.0, 200 mM NaCl). The pellet was then resuspended in the Tris buffer and equal protein content was used for the next step. The isolated mitochondria were then solubilized by addition of Styrene Malic acid (SMA 2:1; Polyscope Polymer) to a final concentration of 0.5%. The solubilized mitochondrial membranes were centrifuged at 100,000 g at 4 °C for 45 min to remove the insoluble fraction and produce a purified SMA lipid particle (SMALP). SMALPs containing GFP-BAK was subsequently enriched by GFP-Trap® magnetic beads (ChromoTek). The purified proteins were analyzed by immunoblotting.

#### Confocal imaging for optogenetic measurements

For measuring the recruitment of BAX after light-induced (optogenetic) BAK activation, cells were transfected with 50 ng pCRY2-mCherry-BAK, 50 ng pCIBN-mCherry-BAK and 50 ng pEGFP-A206K-BAX-C1 (see key resources table). Cells were kept in dark after transfection. The mitochondria were either visualized using MitoTracker™ Deep Red (Thermo Fisher M22426) staining (100 nM) or using transfection with 100 ng mTagBFP2-Tomm20 (see key resources table). Confocal imaging was performed on a TCS SP8 (Leica Microsystems) inverse confocal laser scanning microscope equipped with a PL Apo 63x/1.40 Oil CS2 objective, a 405 nm laser and a tunable white light laser (470 – 670 nm). Fluorescence emission was detected using HyD SMD detectors. Before imaging, the growth medium was changed to 200 μl of phenol-red free DMEM and cells were maintained in a humidified incubator chamber (Ludin IceCube) with 5% CO2. Individual z-stacks were acquired with 2.5 min interval for approx. 60 min. Maximum intensity z-projection was performed using ImageJ and individual time points before and after BAK foci formation were chosen as representative images.

#### BAX and BAK recruitment to GUVs

GUVs with a lipid composition of EPC:CL 7:3 (mol:mol) were prepared by electro-formation as described above. 80 μl of the GUVs suspension was added to a solution of PBS buffer mixed with Alexa 555 soluble dye and 100 nM BAK-Atto 488 in Lab-Tek 8-well chamber slides (NUNC) to a final volume of 200 μl. The samples were heated to 42 °C for 30 min to activate BAK. After cooling the samples to room temperature, 100 nM BAX-Atto 655 was added and incubated for 30 min. Binding of BAK and BAX to the GUVs was measured at individual incubation steps using confocal microscopy. Permeabilization of the GUVs is indicated by the presence of soluble Alexa 555 inside the GUVs. Radial profiles of background-corrected normalized integrated fluorescence intensities were measured for individual GUVs using the Radial Profile Plot Plugin of ImageJ (https://imagej.nih.gov/ij/plugins/radial-profile.html). Radial profile intensities were measured and fit with a Gaussian fitting for n=7 individual GUVs from n = 3 independent experiments.

#### mtDNA release

To detect the release of mitochondrial DNA from mitochondria during apoptosis, WT, BAX KO and BAK KO U2OS cells were seeded in a μ-Dish^35 mm, high^ Grid-500 Glass Bottom chamber (IBIDI) and transfected with 100 ng pcDNA3_Smac1-60_mCherry and pEGFP-Tfam-GFP (see key resources table) 16 h before the experiment. Confocal microscopy images were acquired to identify Smac-mCherry and Tfam-GFP double transfected cells within the coordinate system (Figure 7A) using an LSM 710 microscope (Carl Zeiss) equipped with a C-Apochromat 40x/1.2 water immersion objective (Zeiss). Images were acquired every 2 minutes after apoptosis induction with 1 μM ABT-737, 1 μM S63845 and 10 μM QVD-OPh. The time point of Smac-mCherry release from mitochondria was used to set the start point for kinetic measurements. For the experiments with the BAX(T182I) mutant, BAX/BAK DKO U2OS cells were transfected with Smac-mCherry, TFAM-GFP and Halo-BAX WT or Halo-BAX(T182I) (see key resources table) and imaged the following day with an Echo Revolve fluorescence microscope (RVL-100-M). Cells were fixed by incubation with 4% paraformaldehyde in DMEM and washed three times with PBS after specific time points of Smac release (5-60 min). For the immunostaining of the mitochondria, the cells were permeabilized with 0.25% Triton-X in PBS for 8 min, washed three times for 5 min with PBS and incubated in Blocking Buffer (1% BSA in PBS) for 45 min. Immunostaining was performed with an α-Tomm20 antibody (1:100 dilution in Blocking Buffer, see key resources table) for 45 min. Cells were washed three times for 5 min with PBS and incubated with an Alexa Fluor 633-labelled anti-rabbit secondary antibody (1:200 diluted in Blocking Buffer, see key resources table) for 45 min and washed three times for 5 min with PBS. Images were acquired using an LSM 780 NLO or LSM 880 (Zeiss) equipped with a 63x/1.2 water immersion objective (Zeiss) and an Airyscan module (Zeiss). The cells chosen before by confocal microscopy were relocated using the coordinate system position (Figure 7A). Image analysis was done using ZEN software (Zeiss) and ImageJ. The percentage of cells with mtDNA release was assessed by visual inspection of non-colocalizing Tfam-GFP (green) and Tomm20 (magenta) signals.

#### Flow cytometry

BAX/BAK DKO HCT116 cells were seeded in a 12-well plate and transfected with 300 ng mEGFP-BAK (see key resources table) 16h before the experiment. Apoptosis was induced by incubating the cells with 1 μM STS for one to eight hours. Cells without induction of apoptosis were used as a control. Supernatant and cells were collected, washed with PBS and incubated in 100 nM TMRE in DMEM for 30 min at 37°C in the dark. Cells were pelleted, washed three times with PBS and resuspended in PBS. Cells were analyzed using a FACSCanto flow cytometer (BD Biosciences).

#### Coculture experiments

Single cell solutions of WT, BAK KO and BAX KO SVEC cells were prepared in complete cell media containing 106 cells/mL. While in suspension, the cells were treated with 5 μM ABT-737, 5 μM S63845 and 10 μM QVD for 3 hours. Next, the cells were washed twice by centrifugation at 300 x g for 5 minutes and resuspended in 50 mL PBS. After washing, the treated or untreated cells were plated in 6 well plate (2.5^∗^10ˆ5 cells/well) followed by addition of single cell suspension of spleenocytes (5^∗^10ˆ5 per well) and incubated for 24 hours at 37°C with 5% CO_2_. The cocultured samples were then collected and stained with a viability dye for 30 minutes at room temperature. The viability staining was then followed by two washing steps with PBS and FC-receptor blocking for 10 minutes prior to antibody incubation for 30 minutes at 4°C (see key resources table). Stained cells were then washed and fixed with BD CellFIX (340181, BD Biosciences) for 30 minutes at 4°C before the samples were washed and resuspended in FACS buffer (5% FBS and 2 mM EDTA in PBS). Detailed information regarding reagents used in this procedure can be found in Table 3. All flow cytometry data was acquired using “LSR-Fortessa Analyzer” and analysed with FlowJo software.

### Quantification and statistical analysis

Statistical details of the experiments are provided in the respective figure legends. Graphs were plotted using GraphPad Prism and Origin. Statistical significance was tested as described in the figure legends. Unless indicated differently, data are representative for three independent experiments and were analyzed in a blinded and, if applicable, automated fashion to avoid confirmation and observer bias.

## Data Availability

• Original western blot images and microscopy data have been deposited as raw tiff files at Mendeley and are publicly available as of the date of publication. The DOI is listed in the key resources table. Additional data reported in this paper are available from the lead author upon request. • The software code for the stoichiometry analysis software (SAS) (Danial et al., 2022) is available from github as indicated in the key resources table. DOIs of additional software used are listed in the key resources table. • Any additional information required to reanalyze the data reported in this paper is available from the lead contact upon request.

## Supplemental information

Document S1. Figures S1–S8

Document S2. Article plus supplemental information
