# Supplementary material for: The interplay between BAX and BAK tunes apoptotic pore growth to control mitochondrial-DNA-mediated inflammation
Source: Mol Cell. 2022 Mar 3;82(5):933–949.e9. doi: 10.1016/j.molcel.2022.01.008 (PMC8901441; doi:10.1016/j.molcel.2022.01.008)
Supplement: Document S1. Figures S1–S8 [file mmc1.pdf]

Molecular Cell, Volume *82*

Supplemental information

The interplay between BAX and BAK tunes apoptotic pore growth to control mitochondrial-DNA-mediated inflammation

Katia Cosentino, Vanessa Hertlein, Andreas Jenner, Timo Dellmann, Milos Gojkovic, Aida Peña-Blanco, Shashank Dadsena, Noel Wajngarten, John S.H. Danial, Jervis Vermal Thevathasan, Markus Mund, Jonas Ries, and Ana J. Garcia-Saez

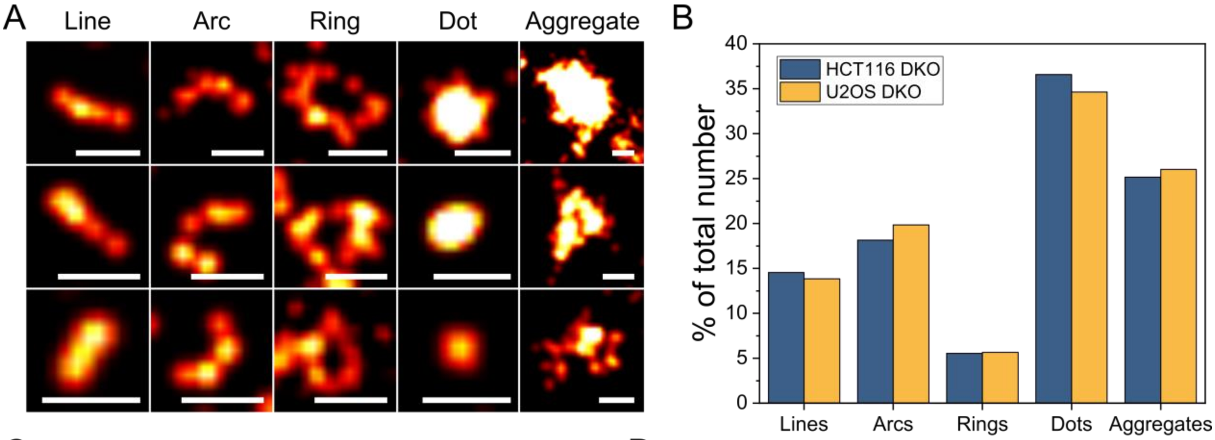

C

	HCT116 DKO	U2OS DKO
Line length	109 nm ± 32 nm	107 nm ± 37 nm
Arc length	103 nm ± 36 nm	101 nm ± 33 nm
Perimeter	106 nm ± 40 nm	104 nm ± 31 nm
Arc radius	24 nm ± 8 nm	25 nm ± 9 nm
Ring radius	18 nm ± 9 nm	17 nm ± 7 nm

D

	GFP-BAK	GFP-BAX
Line length	109 nm ± 32 nm	214 nm ± 80 nm
Arc radius	24 nm ± 8 nm	73 nm ± 30 nm
Ring radius	18 nm ± 9 nm	34 nm ± 7 nm

Figure S1: BAK structures in other cell lines have similar structure distribution and parameter sizes. Related to Fig. 2. A) Gallery of BAK structures in apoptotic BAX/BAK DKO U2OS cells. Scale bar 100 nm. B) Percentage distribution of the different BAK structure types found in BAX/BAK DKO HCT116 (n=12) and U2OS (n=7) cells. Number of lines (minimum 254), arcs (minimum 363), rings (minimum 109), dots (minimum 647) and aggregates (minimum 472). C) Comparison of the average size of mEGFP-BAK structures in BAX/BAK DKO HCT116 and U2OS cells. D) Comparison of mEGFP-BAK and mEGFP-BAX average size structures detected in mEGFP-BAX BAX/BAK DKO Hela (Salvador-Gallego et al., 2016) and mEGFP-BAK BAX/BAK DKO HCT cells.

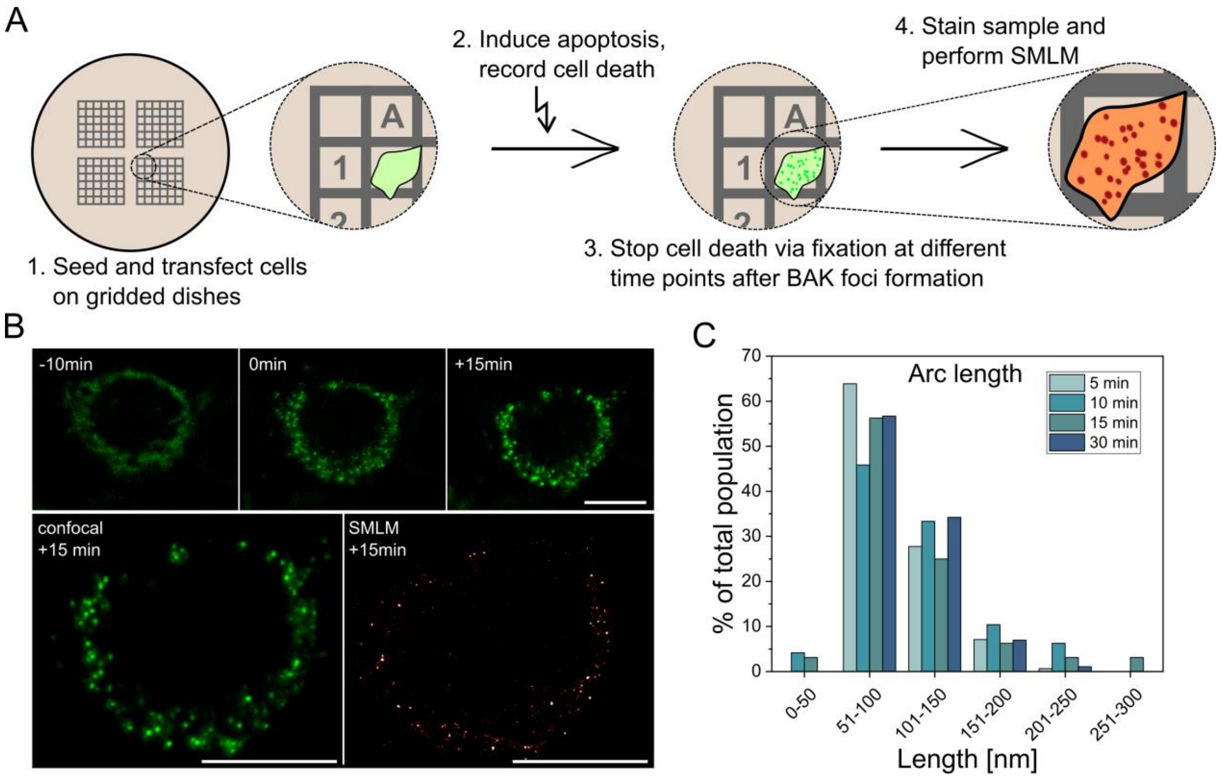

Figure S2: BAK structures evolve over time from linear to ring structures but do not show growth in size. Related to Fig. 2. A) Scheme explaining the principles of correlative fluorescence and super-resolution microscopy. Individual selected mEGFP-BAK transfected cells within the coordinate system were imaged every 2 minutes by confocal microscopy after apoptosis induction with 1 µM ABT-737, 1 µM S63845 and 10 µM qVD-Oph. The time point of first BAK foci appearance was noted and apoptosis progression was stopped by fixation. After immunostaining of mEGFP-BAK with an Alexa647 nanobody, cells were identified using the coordinate system and SMLM imaging was performed. B) Upper panel: Representative confocal fluorescence microscopy images of a BAX/BAK DKO HCT116 cell transfected with mEGFP-BAK and imaged 10 min before foci formation (-10 min), at the time point of first foci appearance (0 min) and 15 min after foci formation (+15 min). Lower panel: Comparison of the same cell imaged with confocal microscopy (left) and SMLM (right). Images are representative of at least 15 independent measurements. Scale bar 10 µm. C) Distribution of the arc length at the different time points after the first foci appearance (minimum number of analysed arcs per time point: 112).

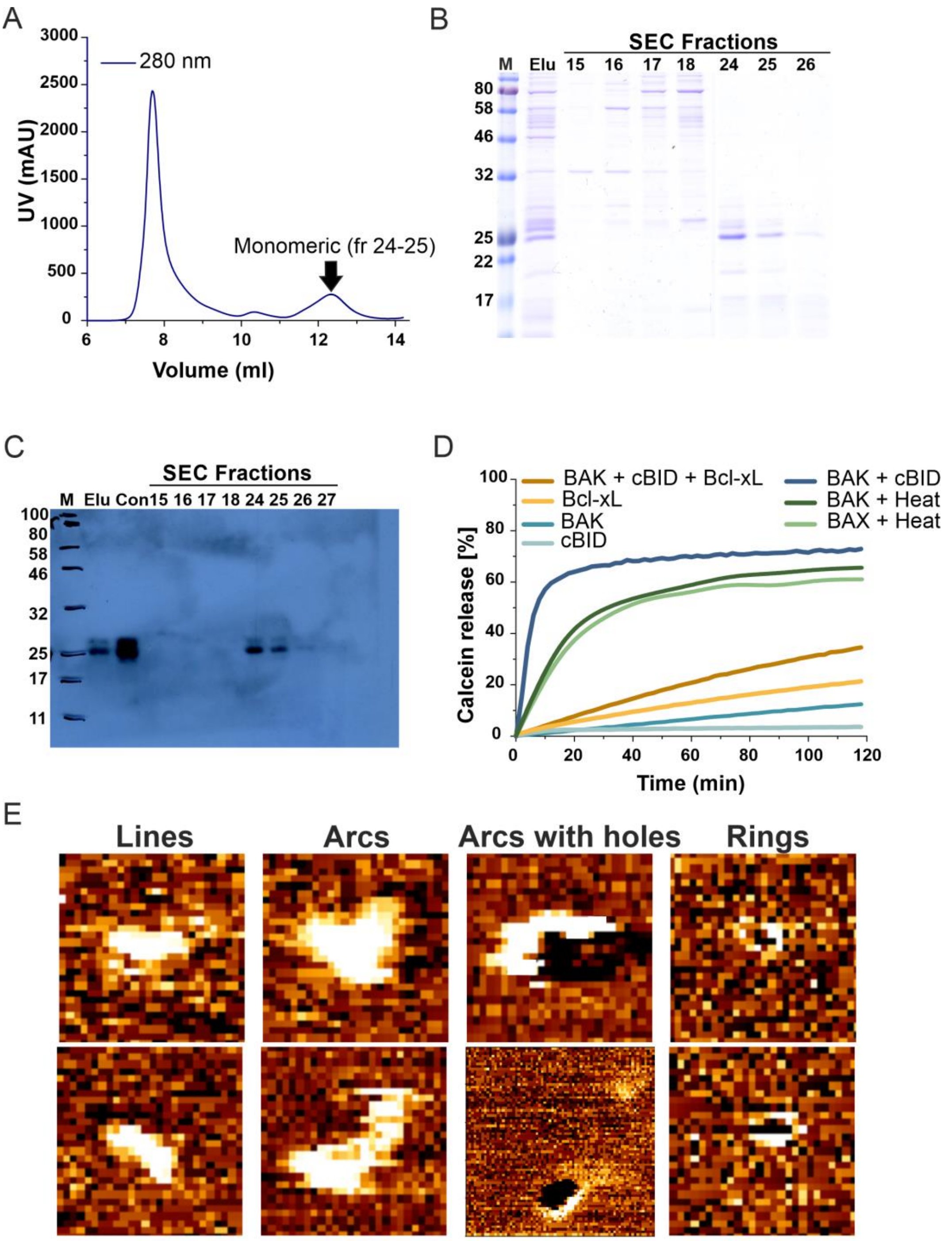

F

BAK Structure	Dimension (nm)
Line length	33.44 ± 3.76
Arc length	5.57 ± 7.481
Arc radius	5.98 ± 3.28
Ring radius	8.12 ± 3.03
Ring external radius	15.98 ± 3.78

Figure S3: Purification, activity characterization, structures and associated membrane pores of full-length BAK. Related to Fig. 3. A) Size exclusion chromatogram of recombinant full-length BAK (BAK I192K, F193S, V196D). The arrow indicates the monomeric fractions. B) SDS-PAGE gel bands (Coomassie brilliant blue staining) and C) western blot indicating the presence of pure BAK (23 kDa). D) Calcein permeabilization assay of purified BAK (200 nM) activated by cBid (20 nM) or heat and inhibited by Bcl-xL (100 nM). LUVs containing a self-quenching/highly concentrated calcein solution were incubated with activated BAK. Release of fluorescent calcein, indicating membrane permeabilization, was monitored over time. Heat-activated BAX (200 nM) was used as a positive control. All data are representative of at least 3 independent experiments. E) Gallery of BAK structures in EPC:CL (80:20 mol %) membranes imaged by atomic force microscopy (AFM). Picture size 100 nm. The full colour height range of the topographs from low (brown-orange) to high (yellow-white) is 2 nm. F) Average size and standard deviation of the different parameters for BAK structures as in E (lines =14, arcs=31 and rings=14). Data were obtained from at least four independent experiments.

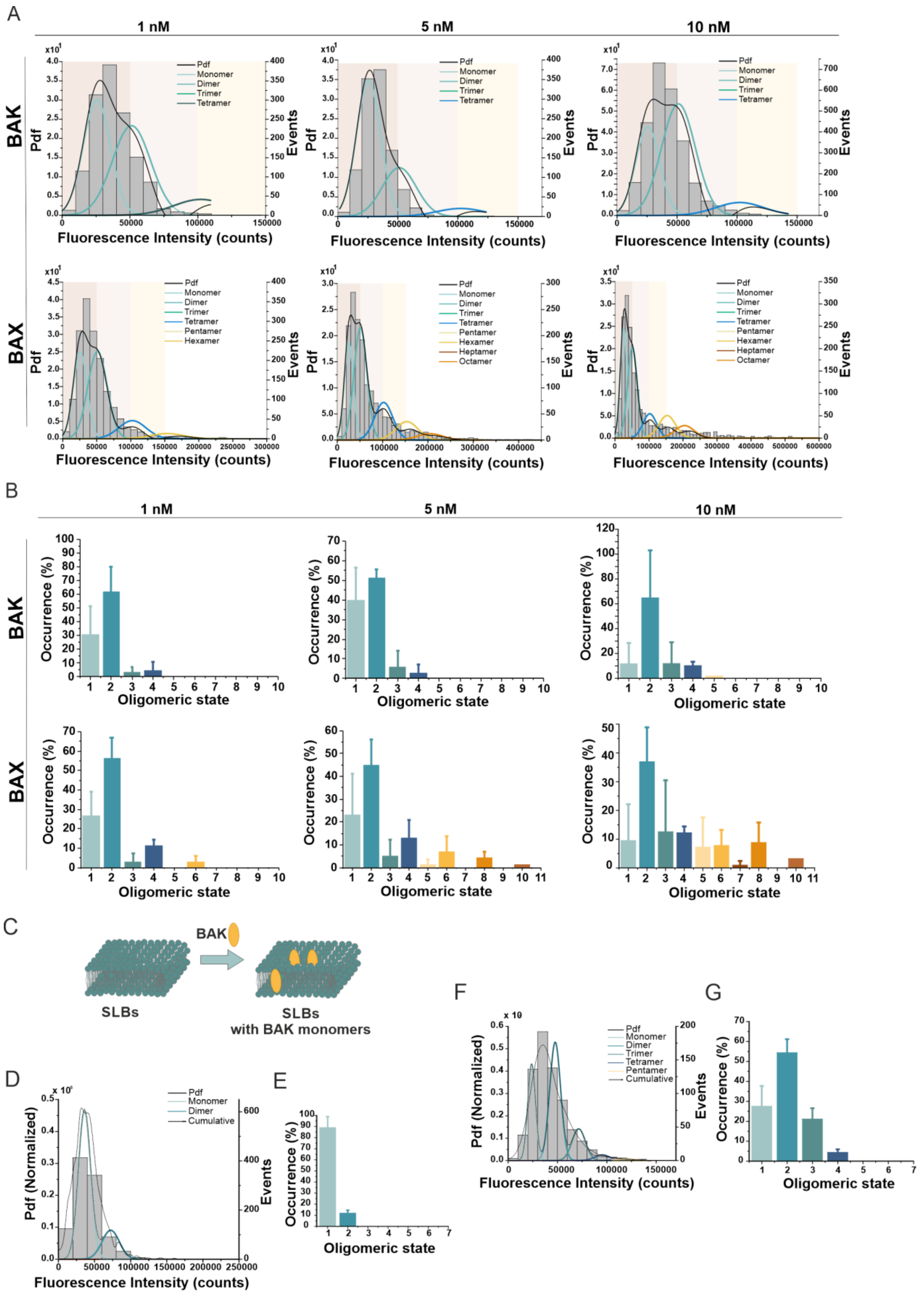

Figure S4: Stoichiometry of BAK oligomers on SLB and activation by cBid. Related to Fig. 3.
A) Representative fluorescence intensity distribution of BAK-488 (upper panel) and BAX-488

(lower panel) oligomers (minimum 1500 particles per condition, from 2 independent experiments) obtained from SLB samples with different concentrations (1, 5 and 10 nM) of protein. The obtained brightness distribution was plotted as a probability density function (Pdf, black) or, alternatively, as a histogram, and fitted with a linear combination of Gaussians to estimate, from the area under each curve, the percentage of occurrence of particles containing n-mer labelled molecules (see the colour code in the graph). The three panels in the background indicate each a 50,000 counts range and are for a visual comparison between BAX and BAK intensity distribution graphs. B) Percentage of occurrence of BAK-488 (upper panel) and BAX-488 (lower panel) different oligomeric species measured from graphs as in A) and calculated as the average value from two different experiments. Data provided are corrected for partial labeling (see STAR Methods). The error bars correspond to the standard deviation from the different experiments. C) Schematic representation of the protocol used for sample preparation in D and E. SLBs were incubated with 1 nM BAK-488 (yellow) and 2 nM cBid (not shown) for 30 minutes at room temperature, washed carefully with buffer and immediately imaged by TIRF microscopy. D) Representative intensity distribution of BAK-488 particles directly added on SLBs as in C). The obtained brightness distribution was plotted as a probability density function (Pdf, black) or, alternatively, as a histogram, and fitted with a linear combination of Gaussians to estimate, from the area under each curve, the percentage of occurrence of particles containing n-mer labelled molecules (see the color code in the graph). E) Percentage of occurrence of different BAK-488 oligomeric species measured from graphs as in D) and calculated as the average value from three different experiments. Due to unspecific interaction of BAK molecules with the SLB glass support, BAK molecules were unable to oligomerize, resulting mainly in monomers and few dimers. F) Representative intensity distribution of BAK-488 particles bound to SLBs prepared from proteoliposomes as in Figure 3, after activation of 1 nM BAK by 2 nM cBid and 1 h incubation at room temperature. G) Percentage of occurrence of different BAK-488 oligomeric species measured from graphs as in F) calculated as the average value from three different experiments. Data provided are corrected for partial labeling. The error bars correspond to the standard deviations from the different experiments.

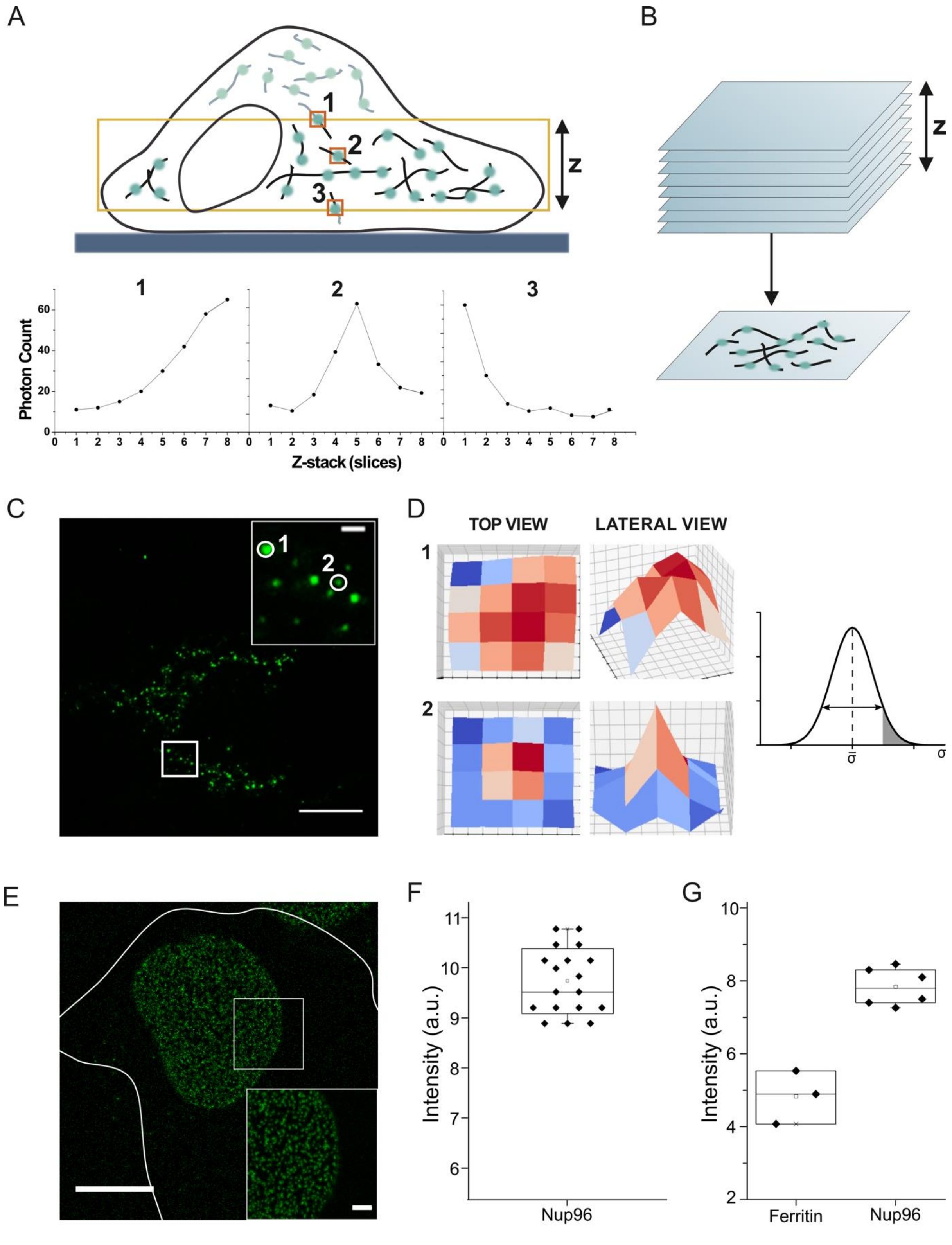

Figure S5: Ratiometric stoichiometry analysis using single-particle photon counting with confocal microscopy. Related to Fig. 4. A) Top panel: Schematic representation of a cell containing mEGFP-BAX or mEGFP-BAK foci (green dots) at mitochondria. A centered z-section of the cell was imaged (indicated by the yellow square) containing individual particles (orange boxes) located at the top z-plane (1), centered in the z-range (2) and at the bottom z-plane

(3). Bottom panel: Intensity of indicated particles 1-3 along the z-axis. Particles 1 and 3 were discarded from the analysis because we could not exclude that their maximum intensity was out of the imaged area. B) Maximum intensity projection of individual z-planes. C) Representative image of maximum intensity z-projection of mEGFP-BAX particles. Inset: Enlarged view of the indicated square with localized regions of interest (circles) containing particles 1 and 2. Scale bar 10 µm, inset scale bar 2 µm D) Left: Representative top and lateral view graphs of the detected fluorescence intensity of the individual fluorescent particles 1 and 2 of C); fluorescence intensity is colour-coded from low (blue) to high (red). Right: Normal distribution of the full width at half maximum (σ) of the fitted 2D Gaussian curves for all detected ROIs. Those ROIs whose σ fell out the 95th percentile of the distribution (grey shaded area) were excluded, as the bigger σ value is likely indicative of the presence of more particles in these ROIs. E) Representative maximum intensity z-projection image of Nup96-mEGFP complexes in a U2OS cell. Inset shows an enlarged view of the indicated square. Scale bar 10 µm, inset scale bar 2 µm. F) Average intensity of Nup96-mEGFP particles obtained from individual cells (dots in box plot). G) Validation of the 32-mer Nup96-mEGFP stoichiometry by ratiometric comparison to the average fluorescence intensity of the 24-mer mEGFP-Ferritin used as an external standard and imaged under the same conditions as Nup96-mEGFP particles.

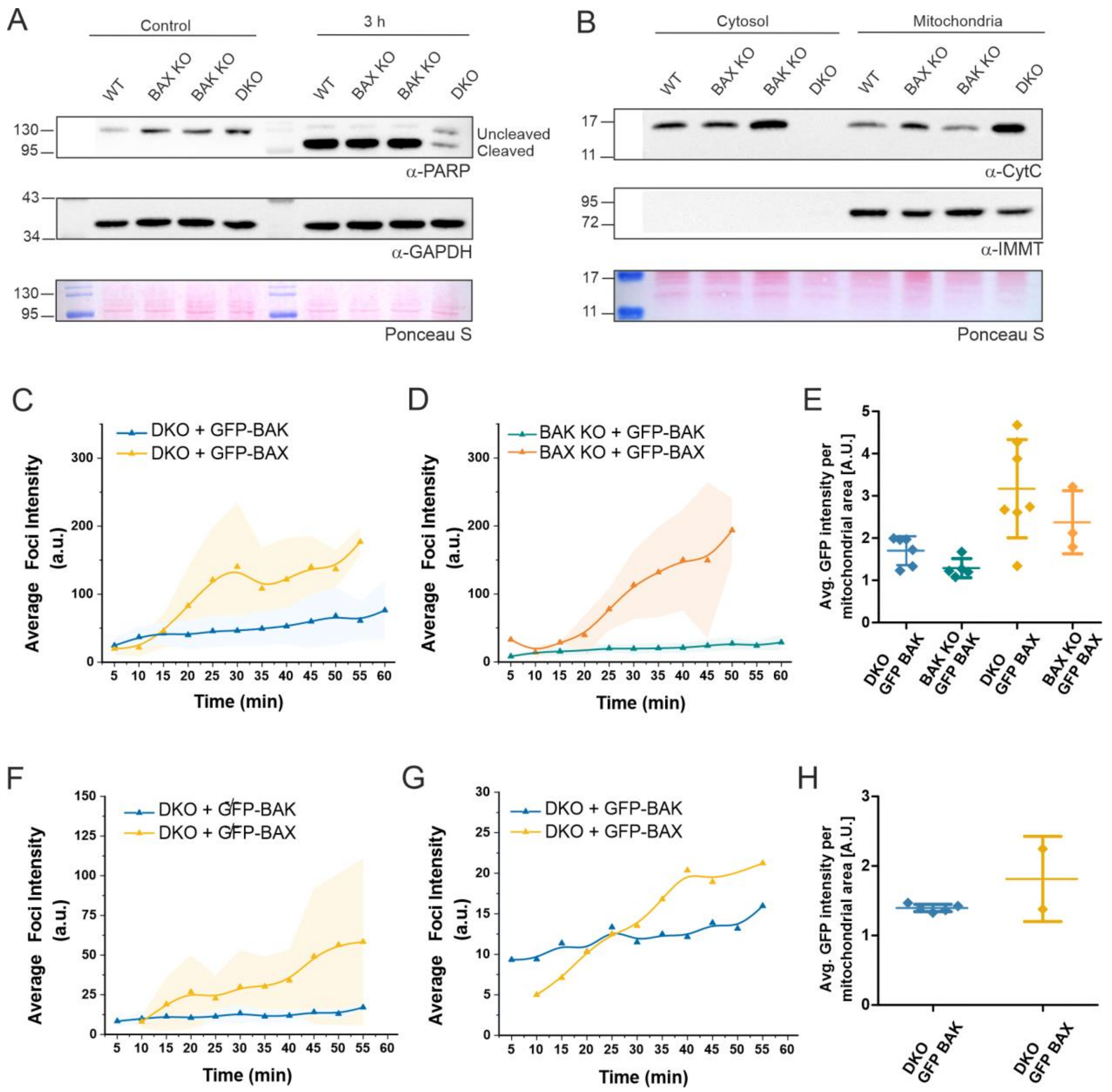

Figure S6: BAX and BAK kinetics of assembly in apoptotic cells by a different apoptosis treatment and in different cell lines. Related to Fig. 4. PARP cleavage (A) and release of cytochrome *c* to the cytosol (B) in WT, BAX KO, BAK KO and BAX/BAK DKO U2OS cells at 3 hours after apoptosis induction with ABT-737, S63845 and qVD-OPh, as typical hallmarks of apoptosis. α-GAPDH and/or Ponceau S staining is shown to control for equal sample loading; α-IMMT immunoblotting was used to control for purity of the cytosolic fraction in B). C) Average mEGFP-BAX (yellow, n° cells=7, minimum average number of foci per time point: 172) and mEGFP-BAK (blue, n° cells=6, minimum average number of foci per time point: 108) foci intensity over time in BAX/BAK DKO U2OS cells transfected with mEGFP-BAX or mEGFP-BAK after apoptosis induction by 1 µM of staurosporine (STS). D) Average mEGFP-BAX foci intensity in BAX KO U2OS cells transfected with mEGFP-BAX (orange, n° cells=3, minimum average number of foci per time point: 32) and for mEGFP-BAK in BAK KO U2OS cells transfected with mEGFP-BAK (green, n° cells=5, minimum average number of foci per time point: 124) after apoptosis induction by 1 µM STS. E) Average mEGFP expression level per mitochondrial area

detected by single-cell fluorescent intensity analysis of mEGFP signal in individual BAX KO, BAK KO or BAX/BAK DKO U2OS cells (individual dots in the plot) transfected with mEGFP-BAX or mEGFP-BAK. F) Average mEGFP-BAX (yellow, n° cells=2) and mEGFP-BAK (blue, n° cells=5) foci intensity over time in BAX/BAK DKO HCT116 cells transfected with mEGFP-BAX or mEGFP-BAK after apoptosis induction by 1 µM ABT-737, 1 µM S63845 and 10 µM qVD-OPh. G) Average foci intensity for mEGFP-BAX (yellow) and mEGFP-BAK (blue) over time in single cells extracted from F) with the same level of expression of mEGFP-BAX and mEGFP-BAK. H) Average mEGFP expression level per mitochondrial area detected by single-cell fluorescent intensity analysis of mEGFP signal in individual BAX/BAK DKO HCT116 cells (individual dots in the plot) transfected with mEGFP-BAX or mEGFP-BAK. In C, D, F, G, foci intensity is not normalized to a calibration standard, but reported in arbitrary units (a.u.). Lines in the graph correspond to the average values from all measured cells and colored areas in the graphs correspond to data variability from single cells.

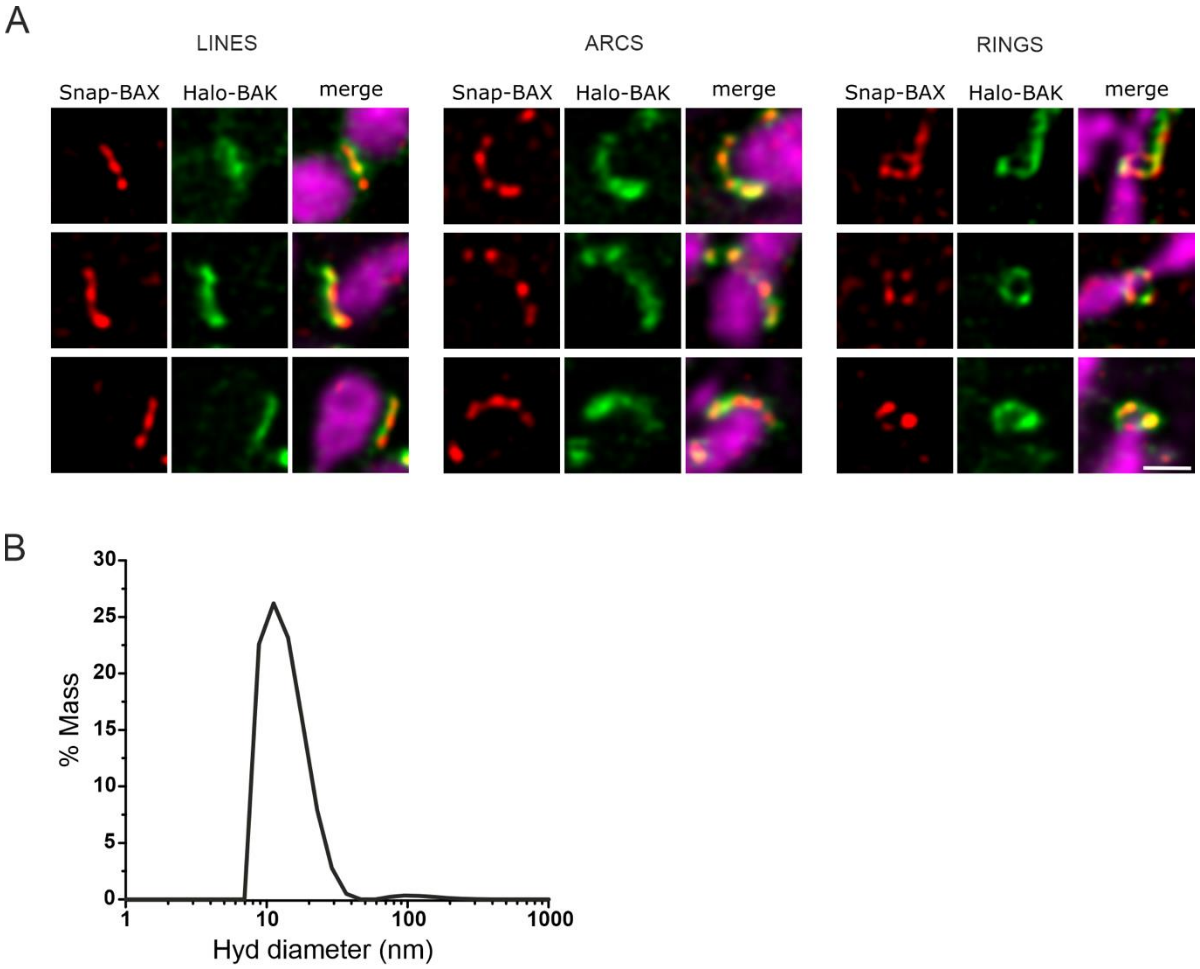

Figure S7: Visualization of BAX-BAK structures by STED microscopy. **Related to Fig. 6.** A) Gallery of line, arc and ring structures formed by BAX and BAK in apoptotic mitochondria acquired by live-cell STED microscopy in BAX/BAK DKO U2OS cell transfected with Snap-BAX (red), Halo-BAK (green) and 4xmt-mTurquoise (magenta, confocal) to stain mitochondria. Apoptosis was induced with 1µM ABT-737, 1 µM S63845 and 10 µM qVD-OPh for 30 min. Scale bar 500 nm. Images show the separated and merged channels of the same structures shown in Figure 6. Images were deconvolved using Huygens Professional and are representative of three independent experiments. B) Dynamic light scattering graph showing the size distribution (hydrodynamic diameter) of SMALPs.

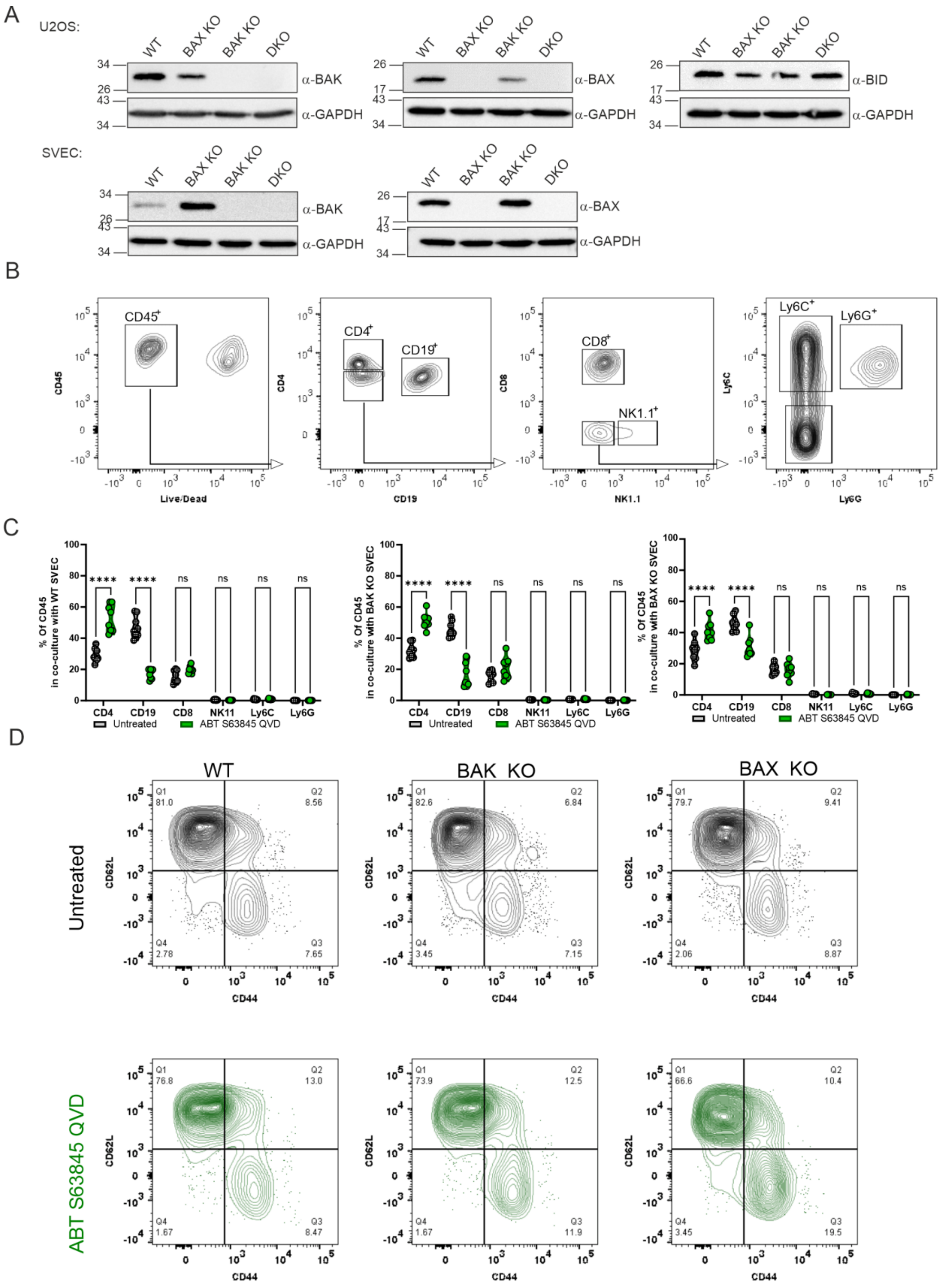

Figure S8: Apoptotic SVEC cells lead to changes in lymphocyte frequencies in a coculture system. Related to Fig. 7. A) Expression levels of endogenous BAX, BAK and BID in WT, BAK KO, and BAX KO U2OS (upper) and SVEC (lower) cells by western blot. α-GAPDH was used to control for equal sample loading. B) Examples of gating strategies used to define different cellular subtypes. C) Frequencies of different cell types in the coculture with splenocytes and

SVEC cells either untreated or pre-treated with ABT-737, S63845 and QVD for 3 hours. D) Representative flowcytometry plots demonstrating changes in CD62L and CD44 in CD4 T-cells in coculture system with either WT, BAK KO or BAX KO SVEC cells. 2way ANOVA and Sidak multiple comparison test was used for the statistical analyses. N= 8 spleen donors. *= $p < 0.05$, **= $p < 0.01$, ***= $p < 0.001$, **** = $p < 0.0001$.
